# The genetic architecture of hip shape and its role in the development of hip osteoarthritis and fracture

**DOI:** 10.1093/hmg/ddae169

**Published:** 2024-11-22

**Authors:** Benjamin G Faber, Monika Frysz, Jaiyi Zheng, Huandong Lin, Kaitlyn A Flynn, Raja Ebsim, Fiona R Saunders, Rhona Beynon, Jennifer S Gregory, Richard M Aspden, Nicholas C Harvey, Claudia Lindner, Timothy Cootes, David M Evans, George Davey Smith, Xin Gao, Sijia Wang, John P Kemp, Jonathan H Tobias

**Affiliations:** Musculoskeletal Research Unit, Learning and Research Building, University of Bristol, Southmead Hospital, Bristol BS10 5NB, United Kingdom; Medical Research Council Integrative Epidemiology Unit, Oakfield House, University of Bristol, Bristol BS8 2BN, United Kingdom; Musculoskeletal Research Unit, Learning and Research Building, University of Bristol, Southmead Hospital, Bristol BS10 5NB, United Kingdom; Medical Research Council Integrative Epidemiology Unit, Oakfield House, University of Bristol, Bristol BS8 2BN, United Kingdom; CAS Key Laboratory of Computational Biology, Shanghai Institute of Nutrition and Health, University of Chinese Academy of Sciences, Chinese Academy of Sciences, 320 Yueyang Road, Shanghai 200031, China; Department of Endocrinology and Metabolism, Zhongshan Hospital, Fudan University, 111 Yixueyuan Road, Xuhui District, Shanghai 200031, China; Fudan Institute for Metabolic Diseases, Fudan University, Shanghai 200032, China; Mater Research Institute, The University of Queensland, 37 Kent Street, Woolloongabba, Brisbane QLD 4102, Australia; Division of Informatics, Imaging and Data Sciences, The University of Manchester, Oxford Road, Manchester M13 9PL, United Kingdom; Centre for Arthritis and Musculoskeletal Health, University of Aberdeen, Kings College, Aberdeen AB24 3FX, United Kingdom; Musculoskeletal Research Unit, Learning and Research Building, University of Bristol, Southmead Hospital, Bristol BS10 5NB, United Kingdom; Centre for Arthritis and Musculoskeletal Health, University of Aberdeen, Kings College, Aberdeen AB24 3FX, United Kingdom; Centre for Arthritis and Musculoskeletal Health, University of Aberdeen, Kings College, Aberdeen AB24 3FX, United Kingdom; Medical Research Council Lifecourse Epidemiology Centre, University of Southampton,Tremona Road, Southampton SO16 6YD, United Kingdom; NIHR Southampton Biomedical Research Centre, University of Southampton, Tremona Road, Southampton SO16 6YD, United Kingdom; University Hospital Southampton NHS Foundation Trust, Southampton General Hospital, Tremona Road, Southampton SO16 6YD, United Kingdom; Division of Informatics, Imaging and Data Sciences, The University of Manchester, Oxford Road, Manchester M13 9PL, United Kingdom; Division of Informatics, Imaging and Data Sciences, The University of Manchester, Oxford Road, Manchester M13 9PL, United Kingdom; Institute for Molecular Bioscience, The University of Queensland, 306 Carmody Road, Brisbane St Lucia QLD 4067, Australia; The University of Queensland Diamantina Institute, The University of Queensland, Brisbane St Lucia, QLD 4072, Australia; Medical Research Council Integrative Epidemiology Unit, Oakfield House, University of Bristol, Bristol BS8 2BN, United Kingdom; Department of Endocrinology and Metabolism, Zhongshan Hospital, Fudan University, 111 Yixueyuan Road, Xuhui District, Shanghai 200031, China; Fudan Institute for Metabolic Diseases, Fudan University, Shanghai 200032, China; CAS Key Laboratory of Computational Biology, Shanghai Institute of Nutrition and Health, University of Chinese Academy of Sciences, Chinese Academy of Sciences, 320 Yueyang Road, Shanghai 200031, China; Medical Research Council Integrative Epidemiology Unit, Oakfield House, University of Bristol, Bristol BS8 2BN, United Kingdom; Mater Research Institute, The University of Queensland, 37 Kent Street, Woolloongabba, Brisbane QLD 4102, Australia; Frazer Institute, The University of Queensland, 37 Kent Street, Woolloongabba, Brisbane QLD 4102, Australia; Musculoskeletal Research Unit, Learning and Research Building, University of Bristol, Southmead Hospital, Bristol BS10 5NB, United Kingdom; Medical Research Council Integrative Epidemiology Unit, Oakfield House, University of Bristol, Bristol BS8 2BN, United Kingdom

**Keywords:** genome wide association study, osteoarthritis, hip shape, hip fracture, Mendelian randomisation

## Abstract

**Objectives:**

Hip shape is thought to be an important causal risk factor for hip osteoarthritis and fracture. We aimed to identify genetic determinants of hip shape and use these to assess causal relationships with hip osteoarthritis.

**Methods:**

Statistical hip shape modelling was used to derive 10 hip shape modes (HSMs) from DXA images in UK Biobank and Shanghai Changfeng cohorts (*n*_total_ = 43 485). Genome-wide association study meta-analyses were conducted for each HSM. Two-sample Mendelian randomisation (MR) was used to estimate causal effects between HSM and hip osteoarthritis using hip fracture as a positive control.

**Results:**

Analysis of the first 10 HSMs identified 203 independent association signals (*P* < 5 × 10^−9^). Hip shape SNPs were also associated (*P* < 2.5 × 10^−4^) with hip osteoarthritis (*n* = 26) and hip fracture (*n* = 4). Fine mapping implicated *SMAD3* and *PLEC* as candidate genes that may be involved in the development of hip shape and hip osteoarthritis. MR analyses suggested there was no causal effect between any HSM and hip osteoarthritis, however there was evidence that HSM2 (more obtuse neck-shaft angle) and HSM4 (wider femoral neck) have a causal effect on hip fracture (OR_IVW_ method 1.27 [95% CI 1.12–1.44], *P* = 1.79 × 10^−4^ and OR_IVW_ 0.74 [0.65–0.84], *P* = 7.60 × 10^−6^ respectively).

**Conclusions:**

We report the largest hip shape GWAS meta-analysis that identifies hundreds of novel loci, some of which are also associated with hip osteoarthritis and hip fracture. MR analyses suggest hip shape may not cause hip osteoarthritis but is implicated in hip fractures. Consequently, interventions targeting hip shape in older adults to prevent hip osteoarthritis may prove ineffective.

## Graphical Abstract

**Graphical Abstract:**
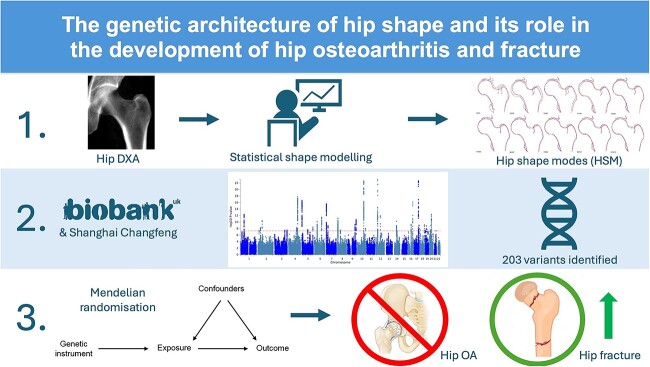

## Introduction

Hip shape varies between individuals and across populations [1]. Certain hip shape variations are associated with disease. For example, an aspherical femoral head (cam morphology) [2–4], reduced acetabular coverage [5] and wider femoral neck [6] have been associated with hip osteoarthritis and a more obtuse femoral-neck angle (neck-shaft angle) and wider femoral neck have been associated with hip fracture [7]. Therefore, understanding the genetic aetiology of hip shape variation provides not only important biological information but also opportunities to better understand disease aetiology. Previous genome-wide association studies (GWAS) have looked at hip shape highlighting the role of endochondral and bone pathways in its development [8, 9]. GWAS can also provide genetic instruments for traits that be used in Mendelian randomisation (MR) to estimate causal effects. This method is less susceptible to confounding and has been likened to a natural randomised control study that does not require years of follow up [10–12].

The observational studies showing an association between hip shape and hip osteoarthritis are prone to confounding [13, 14]. For example, it has been reported that young athletes are at increased risk of cam morphology [4, 15, 16]. However, this observed association might result from confounding due to excess physical activity causing hip osteoarthritis, rather than cam morphology. Differentiating between a causal and confounded association is important as surgical correction of hip shape has been proposed to prevent or delay the onset of hip osteoarthritis and trials are ongoing [17, 18]; it has been difficult to test this hypothesis through randomised control trials due to the slow onset of hip osteoarthritis [13].

We recently used MR to investigate whether a more aspherical femoral head (i.e. cam morphology) has a causal effect on hip osteoarthritis prevalence, with null findings [19]. However, it’s possible other aspects of hip shape have a causal effect on the risk of hip osteoarthritis. Statistical shape modelling provides a holistic measure of hip shape by deriving principal components, also termed hip shape modes (HSMs), from points placed around the joint outline on images [5, 20]. In a prior study using UK Biobank (UKB) data, 7 out of the 10 HSMs, which collectively explained the majority (> 85%) of hip shape variance, were associated with both radiographic and hospital diagnosed hip osteoarthritis, as well as total hip replacement [21], suggesting these imaging phenotypes are clinically relevant. The automated approaches used to derive these HSMs from substantial numbers of participants provides an opportunity to conduct GWAS of multiple HSMs across different cohorts, which would in turn provide well powered genetic instruments that together represent a comprehensive proxy for hip shape, suitable for use in MR analyses.

**Figure 1:**
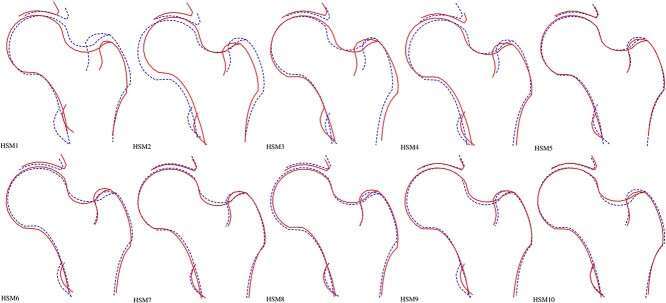
Visual representation of the first 10 hip shape modes. Each hip shape mode (HSM) represents statistically independent (orthogonal) variation in hip shape captured by hip DXA. HSM1 explains the most variation and each HSM there after explains less variation. The HSMs are plotted here where the dotted line represents the hip shape comprised of - 2 standard deviation variation away from the mean and solid line represents + 2 standard deviation hip shape away from the mean. Each participant in this study is given a score for each HSM which represents where their hip shape lies within each mode.

Null findings in MR studies, such as our previous study of cam morphology, may reflect the lack of suitably powered genetic instruments. One way to assess the precision and power of genetic instruments for an exposure such as hip shape is to examine their relationships with another disease for which a causal effect is thought to exist. If a causal effect is found between the genetic instruments and one condition but not another, this makes it less likely the null findings result from inaccurate or under-powered genetic instruments. Hip fracture provides a good positive control for hip osteoarthritis given that measures of hip geometry, such as femoral neck width, are well established causal risk factors for hip fracture in both observational [7] and MR studies [22].

In this study, we aimed to conduct a GWAS meta-analysis of the top 10 HSMs, in UKB and Shanghai Changfeng (SC) cohorts, where these HSM have been derived using our novel automated point placement method [1]. Single nucleotide polymorphisms (SNPs) associated with hip shape were then used as genetic instruments in two-sample MR analyses evaluating whether hip shape is causally related to either hip osteoarthritis or hip fracture.

## Results

### Genome-wide association studies

**Figure 2:**
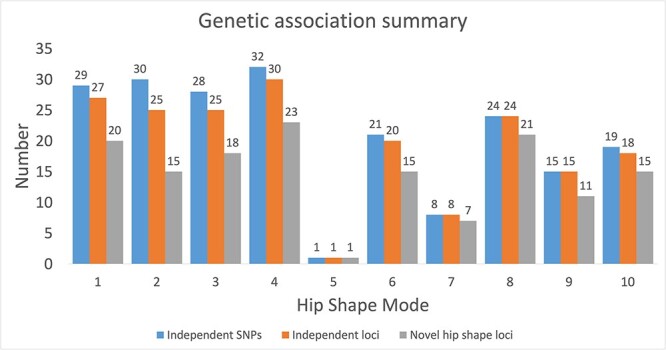
Genetic associations for each hip shape mode. Independent single nucleotide polymorphisms (SNPs) are those that are conditionally independent as obtained by GCTA-COJO. Independent loci are defined by the closest gene. Novel hip shape loci are those further than 1 Mb from previously published SNPs associated with hip shape.

For each HSM [1–10] (Fig. 1) a GWAS meta-analysis was conducted in 43 485 participants (20 580 (47%)/22 905 (53%) male/female) across two studies (UKB *n* = 38 175 & SC *n* = 5310) (see Supplementary Figs. 1–10 for Manhattan plots). The top SNP for each HSM was selected to illustrate effect estimate differences between the cohorts (Supplementary Fig. 11). UKB participants were on average heavier and taller than the SC participants, but their ages were similar (Table 1). Furthermore, the mean HSM scores varied between the sexes and between UKB and SC (Table 1). A total of 131 independent loci were associated with at least one HSM in the random effects meta-analyses (Fig. 2, Supplementary Tables 1–10). These loci were identified from 203 independent SNP associations at study-wide significance (*P* < 5.0 × 10^−9^). Of nine previously identified HSM associated SNPs [9], six showed genome-wide significant associations in the present study (Supplementary Table 11). All five SNPs previously reported to be associated with hip geometry in a previous GWAS [8] showed evidence of association with HSMs, three at genome-wide significance (Supplementary Table 11). SNP heritability for the meta-analysed HSMs, as measured by linkage disequilibrium score regression (LDSC), was between 14%–21%, apart from HSM5, which had a lower SNP heritability of 6% (Table 2). SNP heritability estimates within each cohort, calculated with LDSC, were broadly similar (see Supplementary Table 12). Genomic inflation of the HSM GWAS was low (λ = 0.86–0.94) likely due to the conservative random-effects model used (Supplementary Table 12). 14 SNPs showed at least suggestive evidence of colocalisation with messenger RNA (mRNA) expression in human joint tissue (Supplementary Table 13). Using fixed effect meta-analyses yielded a similar number of associated SNPs to the random effects model (Supplementary Table 14). Given ancestral differences in hip shape and potential genetic heterogeneity we elected to base subsequent analyses on the random effects meta-analyses.

**Table 1:** Descriptive characteristics of study participants, combined and stratified by sex.

	UK Biobank			Shanghai Changfeng		
	Male Mean (range, SD)	Female Mean (range, SD)	Combined Mean (range, SD)	Male Mean (range, SD)	Female Mean (range, SD)	Combined Mean (range, SD)
Age	64.5 (45–81, 7.6)	63.1 (45–82, 7.4)	63.8 (45–82, 7.5)	64.5 (45–81, 9.5)	62.6 (46–88, 9.2)	63.4 (46–96, 9.4)
Height	177.3 (150–204, 6.6)	163.7 (135–196, 6.4)	170.2 (135–204, 9.4)	168.0 (143–189, 6.1)	156.8 (132–185, 5.9)	161.6 (132–189, 8.2)
Weight	83.3 (47–171, 13.4)	68.2 (34–169, 12.8)	75.4 (34–171, 15.1)	69.2 (39–107, 9.8)	59.2 (35–106, 9.1)	63.5 (35–107, 10.6)
HSM1	−0.31 (−4.56–3.57, 1.0)	0.28 (−3.78–3.88,0.9)	0.00 (−4.56–3.88, 1.0)	−0.85 (−3.80–2.22, 0.94)	−0.36 (−4.15–2.70, 0.96)	−0.57 (−4.15–2.70, 0.98)
HSM2	0.02 (−4.53–4.48, 1.0)	−0.01 (−4.69–4.19, 1.0)	0.00 (−4.69–4.48, 1.0)	−0.37 (−4.16–3.95, 1.01)	−0.47 (−4.96–3.30, 1.11)	−0.43 (−4.96–3.95, 1.07)
HSM3	0.32 (−3.62–4.26, 1.0)	−0.30 (−4.10–4.00, 0.9)	0.00 (−4.10–4.26, 1.0)	1.13 (−2.28–4.01, 0.94)	0.83 (−2.45–4.05, 0.95)	0.96 (−2.45–4.05, 0.96)
HSM4	0.14 (−3.83–3.99, 1.0)	−0.13 (−4.38–3.99, 1.0)	0.00 (−4.38–3.99, 1.0)	0.15 (−2.90–4.36, 0.92)	−0.003 (−3.07–3.96, 0.98)	0.06 (−3.07–4.36, 0.96)
HSM5	−0.41 (−4.52–4.38, 0.9)	0.04 (−4.24–3.42, 1.1)	0.00 (−4.52–4.38, 1.0)	−0.46 (−3.62–2.63, 0.91)	−0.02 (−3.44–2.92, 0.92)	−0.21 (−3.62–2.92, 0.94)
HSM6	−0.26 (−4.58–3.88, 1.0)	0.22 (−3.39–4.99, 1.0)	0.00 (−4.58–4.99, 1.0)	0.36 (−2.51–3.46, 0.89)	0.59 (−2.36–3.57, 0.89)	0.49 (−2.51–3.57, 0.90)
HSM7	−0.10 (−4.58–5.05, 1.0)	0.08 (−4.93–4.74, 1.0)	0.00 (−4.93–5.05, 1.0)	−0.23 (−4.90–2.67, 0.91)	0.17 (−2.56–4.03, 0.91)	0.00 (−4.90–4.03, 0.93)
HSM8	0.08 (−4.84–4.55, 1.0)	−0.07 (−4.35–3.96, 1.0)	0.00 (−4.84–4.55, 1.0)	−0.02 (−3.70–3.03, 1.00)	−0.17 (−3.47–3.57, 1.02)	−0.11 (−3.70–3.57, 1.01)
HSM9	0.31 (−3.65–5.03, 1.0)	−0.28 (−4.13–4.54, 0.9)	0.00 (−4.13–5.03, 1.0)	0.24 (−3.16–3.62, 0.96)	−0.14 (−3.46–3.10, 0.97)	0.02 (−3.46–3.62, 0.99)
HSM10	−0.01 (−4.09–3.84, 1.0)	0.01 (−4.12–3.85, 1.0)	0.00 (−4.12–3.85, 1.0)	0.22 (−3.58–3.60, 1.01)	−0.08 (−4.28–3.41, 0.97)	0.05 (−4.28–3.60, 1.00)
**N**	18 646	20 374	38 175	2263	3047	5310

Units are as follows: Age—years, Height—centimetres, weight—kilograms, hip shape modes (HSM)—standard deviations (SDs)

### Gene set analysis

To prioritise genes contributing to each HSM we applied MAGMA gene set analyses using a Bonferroni adjusted p-value threshold (*P* < 2.70 × 10^−6^, 0.05/18824 number of gene sets tested). In total, 30 gene sets were associated with the HSMs (Supplementary Tables 15). Two gene sets showed associations with more than one HSM; the *skeletal morphogenesis* set was associated with HSM1 (Genes *n* = 217 & P 2.98 × 10^−8^), HSM6 (Genes *n* = 217 & P 3.98 × 10^−7^) and HSM10 (Genes *n* = 217 & P 7.83 × 10^−8^), and the *response to growth factor* set was associated with HSM4 (Genes *n* = 662 & P 1.27 × 10^−7^) and HSM10 (Genes *n* = 662 & P 7.83 × 10^−8^). Seven gene sets were related to skeletal, bone and connective tissue development, 5 to cartilage formation, 4 to signalling pathways known to affect cartilage (TGF-β and growth factor).

### Genetic correlation

LDSC was used to calculate the genetic correlation between HSM and different disease traits. For hip osteoarthritis, HSM2 (rg 0.16 [95% CI 0.06, 0.26]), HSM3 (0.18 [0.09, 0.27]), HSM6 (−0.17 [−0.26, −0.09]) and HSM7 (0.15 [0.05, 0.24]) showed some evidence of genetic correlation. For hip fracture, HSM2 (0.30 [0.13, 0.46]) and HSM4 (−0.40 [−0.57, −0.23]) showed a moderate genetic correlation (Table 2). The genetic correlations between UKB and SC, and with disease outcomes were assessed for each HSM but in SC were underpowered due to the smaller sample size of SC (Supplementary Table 16). Despite the HSMs being orthogonal there was weak to moderate genetic correlation between them suggesting shared underlying genetic aetiology (Supplementary Fig. 12).

**Table 2:** SNP heritability of the hip shape mode meta-analyses and their genetic correlations with hip osteoarthritis and hip fracture.

	Heritability	Hip Osteoarthritis		Hip Fracture	
HSM	h2 [95%CI]	rg [95% CI]	P-value	rg [95% CI]	P-value
HSM1	0.21 [0.17–0.25]	−0.05 [−0.15, 0.04]	0.30	−0.04 [−0.21, 0.13]	0.68
HSM2	0.21 [0.17–0.26]	0.16 [0.06, 0.26]	1.30 × 10^−03^	0.30 [0.13, 0.46]	4.00 × 10^−04^
HSM3	0.18 [0.15–0.22]	0.18 [0.09, 0.27]	2.00 × 10^−04^	0.13 [−0.01, 0.28]	0.07
HSM4	0.19 [0.15–0.23]	−0.04 [−0.14, 0.05]	0.37	−0.40 [−0.57, −0.23]	3.29 × 10^−06^
HSM5	0.06 [0.04–0.08]	0.03 [−0.11, 0.17]	0.70	0.10 [−0.12, 0.33]	0.37
HSM6	0.19 [0.16–0.22]	−0.17 [−0.26, −0.09]	4.97 × 10^−05^	0.03 [−0.11, 0.16]	0.70
HSM7	0.14 [0.11–0.17]	0.15 [0.05, 0.24]	2.60 × 10^−03^	0.07 [−0.10, 0.25]	0.42
HSM8	0.21 [0.17–0.26]	0.09 [−0.02, 0.20]	0.12	0.08 [−0.07, 0.24]	0.31
HSM9	0.15 [0.12–0.19]	−0.05 [−0.15, 0.05]	0.30	0.07 [−0.10, 0.24]	0.41
HSM10	0.17 [0.14–0.21]	0.03 [−0.07, 0.12]	0.60	0.09 [−0.07, 0.24]	0.28

### Genetic overlap between hip shape and hip osteoarthritis

Conditionally independent HSM-associated SNPs were looked up in a previous GWAS of osteoarthritis traits [23]. 26 SNPs were associated with a HSM (genome wide significance *P* < 5 × 10^−9^) and hip osteoarthritis (Bonferroni adjusted *P* < 2.5 × 10^−4^, to account for the 203 SNPs tested) (Table 3). HSM SNPs also showed an association (*P* < 2.5 × 10^−4^) with osteoarthritis at all sites (*n* = 11), at the knee and/hip (n = 18) and total hip replacement (*n* = 28) (Supplementary Table 17). 19 SNPs were > 1 Mb away from previously implicated hip shape loci and represented novel signals. To understand if the hip shape and osteoarthritis GWAS signals were shared Bayesian colocalisation was conducted which found 18 SNPs colocalised (PP > 80%). In particular, two SNPs showed strong SNP-gene evidence after fine-mapping.

**Table 3:** Hip shape signals shared with hip osteoarthritis and hip fracture.

Hip Shape Novel Loci																		
RSID	CHR	BP	EA	NEA	EAF	HSM	BETA	SE	P	DIR	HET.I2	N	HOA. GO.BETA	HOA.GO.P	HOA Coloc PP	MAGMA Gene	MAGMA P	C.GENE
rs60733781	1	118 726 282	T	C	0.03	HSM2	0.12	0.02	6.47E-10	++	0	43 485	0.15	5.64E-08	0.97	SPAG17	4.28E-03	SPAG17
rs56101873	1	119 474 790	A	G	0.17	HSM7	0.06	0.01	6.58E-11	++	0	43 485	0.04	1.16E-04	0.87	TBX15	1.16E-06	TBX15
rs2884774	1	118 779 972	A	G	0.36	HSM9	−0.05	0.01	4.885E-14	−	0	43 485	0.06	7.70E-11	0.99	SPAG17	1.37E-05	SPAG17
rs2367508	2	33 453 924	A	G	0.50	HSM8	−0.04	0.01	4.982E-10	−	0	43 485	0.03	1.44E-04	0.34	LTBP1	1.91E-05	LTBP1
rs227838	6	44 684 621	A	C	0.58	HSM1	−0.04	0.01	6.5E-10	−	0	43 485	0.04	3.35E-06	0.00	RUNX2	1.42E-02	SUPT3H
rs56115627	6	44 683 049	A	G	0.32	HSM3	−0.05	0.01	1.417E-10	−+	0	43 485	0.06	5.10E-10	0.49	SUPT3H	1.96E-04	SUPT3H
rs12199167	6	44 717 205	T	C	0.35	HSM4	0.05	0.01	7.366E-13	++	0	43 485	0.06	1.54E-08	0.55	RUNX2, SUPT3H	7.32E-08, 4.79E-06	SUPT3H
rs6915627	6	35 128 330	A	G	0.66	HSM10	0.04	0.01	2.418E-09	++	0	43 485	−0.04	1.61E-05	0.88	SCUBE3	0.00085427	TCP11
rs520161	7	28 210 660	T	C	0.29	HSM1	0.04	0.01	1.29E-09	++	0	43 485	0.04	1.17E-05	0.89	GNA12	2.47E-09	JAZF1
rs7011532	8	69 650 165	C	G	0.30	HSM2	0.05	0.01	1.93E-12	++	0	43 485	0.04	4.18E-05	0.97	C8orf34	1.79E-04	C8orf34
rs6996725	8	69 642 205	T	C	0.29	HSM4	0.05	0.01	3.058E-10	++	0	43 485	0.04	1.91E-05	0.96	C8orf34	4.25E-02	C8orf34
rs11784501	8	145 013 315	T	G	0.38	HSM7	−0.05	0.01	2.152E-11	−	0	43 485	0.04	3.78E-06	0.93	PLEC	8.53E-04	PLEC
rs10760442	9	129 383 900	A	G	0.36	HSM10	0.05	0.01	2.033E-10	++	0	43 485	−0.06	8.13E-11	0.99	LMX1B	6.08E-06	LMX1B
rs12901499	15	67 370 445	A	G	0.46	HSM4	0.05	0.01	1.694E-13	++	0	43 485	−0.07	2.83E-15	0.99	SMAD3	2.03E-05	SMAD3
rs34882685	17	59 536 624	A	G	0.26	HSM3	0.05	0.01	1.926E-10	++	0	43 485	0.05	1.08E-05	0.20	TBX4	1.95E-10	TBX4
rs4141079	17	59 531 402	A	C	0.74	HSM4	−0.05	0.01	2.054E-13	−	0	43 485	−0.05	1.29E-07	1.00	TBX4	1.26E-09	TBX4
Hip Shape Known Loci																		
RSID	CHR	BP	EA	NEA	EAF	HSM	BETA	SE	P	DIR	HET.I2	N	HOA.GO.BETA	HOA.GO.P	HOA Coloc PP	MAGMA Gene	MAGMA P	C.GENE
rs6663034	1	103 422 537	A	C	0.29	HSM3	−0.05	0.01	3.75E-12	−	0	43 485	−0.070	1.20E-13	0.19	COL11A1	6.41E-09	COL11A1
rs2169608	1	103 350 118	C	G	0.61	HSM8	−0.12	0.01	2.259E-65	−	0	43 485	0.07	5.19E-13	0.01	COL11A1	3.21E-13	COL11A1
rs3753841	1	103 379 918	A	G	0.62	HSM9	0.06	0.01	5.33E-18	++	0	43 485	0.07	4.03E-14	0.02	COL11A1	1.21E-08	COL11A1
rs1372328	9	119 484 528	T	C	0.46	HSM1	−0.04	0.01	5.77E-10	−	0	43 485	−0.07	9.06E-15	0.99	ASTN2	1.40E-04	ASTN2
rs10983319	9	119 483 466	T	C	0.53	HSM2	0.05	0.01	8.46E-14	++	0	43 485	0.07	7.51E-14	0.99	TBX15	1.24E-05	ASTN2
rs258413	12	28 016 988	T	C	0.58	HSM1	−0.06	0.01	8.12E-24	−	0	43 485	−0.06	1.71E-10	0.00	PTHLH	1.16E-04	KLHL42
rs258413	12	28 016 988	T	C	0.58	HSM2	0.05	0.01	3.99E-13	++	0	43 485	−0.06	1.71E-10	0.00	PTHLH	1.13E-05	KLHL42
rs10843013	12	28 025 196	A	C	0.79	HSM6	0.06	0.01	5.151E-14	++	0	43 485	−0.11	2.91E-24	0.96	CCDC91	1.32E-03	KLHL42
rs10743612	12	28 015 391	A	G	0.24	HSM8	0.06	0.01	2.089E-13	++	0	43 485	0.10	6.39E-22	0.86	PTHLH	2.02E-03	KLHL42
rs7958415	12	27 997 918	T	C	0.39	HSM9	−0.05	0.01	2.815E-12	−	0	43 485	0.04	5.02E-05	0.00	CCDC91	3.34E-14	KLHL42
Hip Fracture Novel Loci																		
RSID	CHR	BP	EA	NEA	EAF	HSM	BETA	SE	P	DIR	HET.I2	N	HipFrac.Beta	HipFrac.P	Hip Frac Coloc PP	MAGMA Gene	MAGMA P	C.GENE
rs12475479	2	233 041 502	A	T	0.26	HSM2	−0.06	0.01	4.96E-14	−	0	43 485	−0.08	4.79E-05	0.09	DIS3L2	2.87E-06	DIS3L2
rs56368105	12	54 393 770	A	G	0.56	HSM4	0.06	0.01	1.6E-18	++	0	43 485	−0.08	1.38E-07	0.10	RP11-834C11.12, HOXC6	2.22E-16, 1.09E-14	RP11-834C11.12
rs736825	12	54 417 576	C	G	0.60	HSM9	0.08	0.01	4.37E-31	++	0	43 485	−0.08	8.87E-08	0.08	HOXC9, RP11-834C11.12, HOXC8, HOXC5, HOXC6	5.83E-15,1.21E-13, 3.79E-11, 1.79E-10, 5.00E-10	RP11-834C11.12
rs11614913	12	54 385 599	T	C	0.43	HSM10	0.05	0.01	1.18E-14	++	0	43 485	0.08	1.30E-07	0.10	HOXC9, HOXC6, RP11-834C11.12, HOXC8, HOXC4	1.76E-12, 7.25E-11, 3.39E-10, 7.27E-10, 1.60E-06	RP11-834C11.12

This table contains those independent SNPs associated with a hip shape mode (*P* < 5 × 10^−9^) and hip osteoarthritis (Bonferroni adjusted *P*-value < 1.7 × 10^−4^, accounting for 290 SNPs tested). Hip osteoarthritis (HOA) results (i.e. HOA.GO.BETA, HOA.GO.P) taken from a previous publication [23]. Hip fracture (HipFrac) results taken from a previous publication [47]. Colocalisation was between the hip shape mode GWAS and HOA and hip fracture GWAS, if the posterior probability is > 0.8 this is strong evidence the two signals are shared. The gene with the lowest p-value for that locus as calculated by MAGMA is given as MAGMA gene with its corresponding P value (MAGMA P). This is compared with the closest gene given in the adjacent column (C.Gene). CHR—Chromosome, BP—Base Pair, EA—Effect Allele, NEA—Non-Effect Allele, HSM—hip shape mode associated with SNP, HSM.BETA—hip shape mode effect, HSM.SE—hip shape mode standard error, HOA BETA—hip osteoarthritis effect (beta), HOA P—hip osteoarthritis P-value, HOA coloc PP—Posterior probability of colocalisation, HipFrac.BETA – hip fracture effect (beta), HipFrac.P—hip fracture *P*-value, Hip.Frac coloc PP—Posterior probability of colocalisation.

Rs12901499, within the *SMAD3* locus, was positively associated with HSM4 (β 0.05, P 1.69 × 10^−13^), describing greater acetabular coverage and narrower femoral neck, and was also found to be protective of hip osteoarthritis (β −0.07, P 2.83 × 10^−15^). In addition, the HSM4 and hip osteoarthritis GWAS signals around rs12901499 colocalised with each other (PP 99%) and the HSM4 GWAS signal colocalised with *SMAD3* mRNA expression in both healthy and degraded human cartilage (PP 98% in both tissues, Supplementary Table 13, Supplementary Figs. 13 and 14).

Rs11784501, within the *PLEC* locus, was negatively associated with HSM7 (β −0.05, P 2.15 × 10^−11^), describing a wider femoral neck, and was also suggested to increase the risk of hip osteoarthritis (β 0.04, P 3.78 × 10^−6^) although this signal did not meet genome-wide significance. Again, these two GWAS signals colocalised (PP 93%) and the HSM7 GWAS signal colocalised with *PLEC* mRNA expression in human synovial tissue (PP 87%, Supplementary Table 13, Supplementary Fig. 15). Rs11784501 was predicted to have a high chromatin state activity in both chondrocytes (chromatin state = 2, encodes a region flanking a transcription starting site, 1 is the highest activity state and 15 is the lowest) and osteoblasts (chromatin state = 1, encodes a variant at a transcription starting site) suggesting a role in altered transcription (Supplementary Table 18).

### Overlap between hip shape and hip fracture

Four HSM-associated SNPs (*P* < 5 × 10^−9^) also showed an association with hip fracture (*P* < 2.5 × 10^−4^, to account for the 203 SNPs tested) (Table 3). We also examined the overlap between genetic influences on hip shape and bone mineral density estimated from heel ultrasound, which has also been reported in UK Biobank [24],and may be a useful proxy measure for hip fracture risk. Sixty-six HSM-associated SNPs (*P* < 5 × 10^−9^) also showed an association with bone mineral density (*P* < 2.5 × 10^−4^, Supplementary Table 17).

Rs12475479 was negatively associated with HSM2 (β −0.06, P 4.96 × 10^−14^), describing a reduced femoral neck-shaft angle and greater acetabular coverage, and was found to be protective of hip fracture (β −0.08, P 4.79 × 10^−5^) although this signal did not meet genome-wide significance. *DIS3L2* was implicated by MAGMA at this locus although colocalisation analyses showed little evidence of shared HSM2 and hip fracture GWAS signals. Three SNPs (rs56368105, rs736825 & rs11614913) on chromosome 12 and in close LD with each other (D′ 0.78–0.98, R^2^ 0.51–0.89) showed positive associations with HSM4 (a narrower femoral neck and larger femoral head, β 0.06, P 1.60 × 10^−18^), HSM9 (wider femoral neck and larger femoral head, β 0.08, P 4.73 × 10^−31^) and HSM10 (wider femoral neck, β 0.05, P 1.18 × 10^−14^). Rs56368105 (β −0.08, P 1.38 × 10^−7^) and rs736825 (β −0.08, P 8.87 × 10^−8^) were protective for hip fracture, whereas rs11614913 was associated with increased risk (β 0.08, P 4.73 × 10^−31^). All three SNPs were predicted to increase transcription in both chondrocytes (chromatin state 1, 2 and 2 respectively) with only rs56368105 increasing transcription in osteoblasts (chromatin state 1) (Supplementary Table 19). MAGMA implicated several genes at this locus including *HOXC9, RP11-834C11.12, HOXC8, HOXC5, HOXC6* and *HOXC4* but none of the signals colocalised with mRNA expression in human tissue.

### Mendelian randomisation

**Figure 3:**
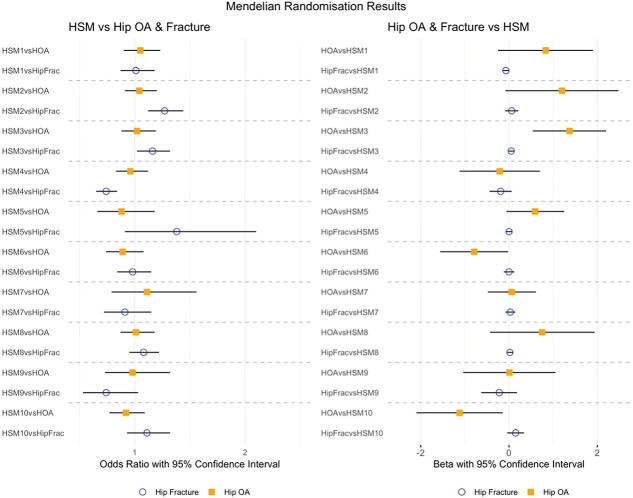
Mendelian randomisation results.Bi-directional causal analyses between hip shape, and hip osteoarthritis and hip fracture.

MR analyses suggested there was no causal effect of any HSM on hip osteoarthritis (Fig. 3, Supplementary Table 18). The mean F-statistics for the genetic instruments of the HSMs ranged from 37 to 56 (Supplementary Tables 1–10) indicating acceptable instrument strength [12]. Reverse MR used 27 genetic instruments for hip osteoarthritis, which had a mean F-statistic of 45 (range 30–100), indicating acceptable instrument strength. Genetic predisposition to hip osteoarthritis was suggested to causally effect HSM3 (cam-type femoral head with bulging of the lateral aspect) (Inverse-variance weighted (IVW) β 1.37 [95% CI 0.54–2.20], P 1.21 × 10^−3^) (Fig. 3, Supplementary Table 19). Sensitivity analyses showed the same direction of effect but with weaker statistical evidence (Supplementary Table 19).

Counter to the results seen for hip osteoarthritis, MR analyses showed strong evidence for HSM2 (more obtuse neck-shaft angle and less acetabular coverage, as represented by the + 2 SD line (Fig. 1)) and HSM4 (less acetabular coverage and wider femoral neck, as represented by the −2 standard deviation (SD) line (Fig. 1)) having a causal effect on hip fracture (IVW OR 1.27 [95% CI 1.12–1.44], P 1.79 × 10^−4^ and OR 0.74 [0.65–0.84], P 7.60 × 10^−6^ respectively) (Fig. 3, Supplementary Table 20). These effects were broadly supported by the sensitivity analyses (Supplementary Table 20). There was weaker evidence that HSM3 (a cam-type femoral head and shorter femoral neck) may also have a causal effect on hip fracture (IVW OR 1.16 [95% CI 1.02–1.32], P 0.02) but the sensitivity analyses showed little evidence to support this. There was little evidence that a genetic predisposition to hip fracture affects any of the HSM modes (Fig. 3  Supplementary Table 20). In addition, the three instruments for hip fracture derived from a previous meta-analysis had acceptable strength (F-statistic range 36–44).

## Discussion

In this study we present the results from a GWAS of 10 orthogonal HSMs that identified 203 conditionally independent SNPs, mapping to 131 loci which is a marked increase on previous hip shape GWAS [9, 25]. In gene set analyses, skeletal, bone and cartilage related pathways showed the strongest evidence of association with hip shape variance. HSMs were genetically correlated with hip osteoarthritis and hip fracture indicating shared underlying genetic aetiology. Of hip shape associated SNPs, 66 were also associated with estimated bone mineral density, 26 with hip osteoarthritis and 4 with hip fracture. MR analyses failed to show evidence of a causal effect of hip shape on hip osteoarthritis but did show a causal effect of hip shape, in the form of a more obtuse neck shaft angle and reduced acetabular coverage, on hip fracture. In addition, there was evidence that a genetic predisposition to osteoarthritis caused a cam-type femoral head to develop.

Understanding the genetic aetiology of hip shape is important given its implication in the aetiology of hip osteoarthritis and hip fracture in observational studies [5, 6, 20, 21, 26]. When investigating the overlap between hip shape loci and both conditions, there were many more hip shape SNPs associated with hip osteoarthritis than hip fracture (26 vs 4). Although this might suggest there is a greater shared genetic aetiology between hip shape and hip osteoarthritis than with hip fracture. We did find 66 shared loci with estimated bone mineral density, which is strongly related to fracture risk [24]. Hip fractures by their nature are normally a consequence of falls (~90%) that themselves are often related to environmental factors [27] meaning they might not be captured well genetically. In terms of specific genes likely to affect both hip shape and either hip osteoarthritis or fracture, *SMAD3* and *PLEC* were the only genes identified through colocalisation between GWAS signals and mRNA expression in human joint tissue and both were associated with hip osteoarthritis. Although the *PLEC* GWAS signal with hip osteoarthritis was only suggestive and did not reach genome-wide significance. *SMAD3* is an important member of the TGF-β pathway which plays an important role in chondrogenesis and has previously been implicated in hip osteoarthritis but was not known to be associated with hip shape [28, 29]. *PLEC* encodes Plectin a large cytoskeleton protein that is important in regulating cellular responses to mechanical stressors and is thought to play a role in the development of osteoarthritis but has not previously been associated with hip shape [30].

The independent SNPs associated with each HSM were used as genetic proxies for the described hip shape variation in MR, to estimate the causal effect of each HSM on hip osteoarthritis and fracture. There was little evidence that any HSM had a causal effect on hip osteoarthritis despite a previous study using the same participants in UKB showing strong observational associations between these HSMs and hip osteoarthritis and total hip replacement [21]. These results counter the hypothesis that moderate alterations in hip shape (e.g. cam morphology or acetabular dysplasia) cause hip osteoarthritis in the general population [17, 31]. However, they align with our recent study, in the same participants, which found no strong evidence of a causal association between cam morphology (based on alpha-angle derived from the same points used to generate HSMs in the present study) and hip osteoarthritis. Importantly the present study had greater power to explore these associations due to higher number of genetic instruments for each HSM (apart from HSM5) as compared to the alpha-angle study which had only 8 genetic instruments [19]. Interestingly, in the previous cam morphology MR study there was strong evidence that a genetic predisposition to hip osteoarthritis caused cam morphology and in the present study the same was seen with HSM3 which resembles a cam-type hip with a bulging of the lateral aspect of the femoral head. This adds evidence to the hypothesis that in part osteoarthritis results from a recapitulation of dysregulated growth [32]. The shared genetic loci and correlations between hip shape and hip osteoarthritis considered alongside these MR findings suggest that other hip shapes such as reduced or increased acetabular coverage represented by HSM1&2 (i.e. acetabular dysplasia and pincer morphology) might have shared underlying genetics with hip osteoarthritis but do not appear to cause hip osteoarthritis.

Our results suggest that, at least within the context of the older adult population studied here, changes in hip shape are unlikely to play a causal role in the development of hip osteoarthritis. These findings have implications for treatments targeting hip shape with a view to delaying onset or slowing the progression of hip osteoarthritis in older adults including surgery that are currently being investigated in randomised trials [18, 33]. On the other hand, rare extremes of hip shape such as developmental dysplasia of the hip which presents in symptomatic children are not well represented in our study population. This is because individuals with such conditions typically undergo hip replacements before the age of 40 years old, making them ineligible for inclusion in the imaging study. Moreover, several rare genetic variants causing more severe forms of hip shape have been identified [34], which are unlikely to have been well proxied by genetic instruments derived from our hip shape GWAS.

To understand if the HSMs were appropriately instrumented by the GWAS results, we also conducted MR analyses between HSMs and hip fracture. These analyses showed strong evidence of a causal association between hip shape and risk of hip fracture. Specifically, HSM2 (more obtuse neck shaft angle and less acetabular coverage) and HSM4 (less acetabular coverage and wider femoral neck) were associated with an increased risk of hip fracture. These findings support the observational results seen in a recent meta-analysis, which reported an elevated risk of hip fracture with increased femoral neck shaft angle and femoral neck width [7]. These findings also align with those of our recent MR study, which found that increased femoral neck width acts causally to increase risk of hip fracture [22]. Taken together, our results suggest that hip shape is a risk factor for hip fracture, supporting the validity and power of these hip shape instruments and adding further weight to the null associations seen for hip osteoarthritis. Furthermore, in both hip osteoarthritis and hip fracture MR analyses only unidirectional effects were seen indicating causal relationships as opposed to bidirectional effects indicating shared genetics.

This study combined two cohorts which evaluated hip shape using high resolution DXA scans, commonly used to screen for osteoporosis risk. Automatically extracting additional hip shape information from DXA scans in terms of predicting hip osteoarthritis and fracture risk is an attractive proposition. Even if hip shape variance is not causally associated with hip osteoarthritis, these measures could still be important predictive factors. The majority of previous hip shape studies have focused on European populations, but in this study we included an East Asian ancestry group, which broadens the generalisability of our findings [7, 35]. However, conducting trans-ancestry GWAS introduces complexity, due to the inherent heterogeneity between populations [36]. In this study we used a conservative random-effects model to take account of this heterogeneity and limit the possibility of spurious false positive results driven by the larger size of UKB. Given the disparity in sample size between UKB and SC, we felt it unwise to explore ethnic differences directly in this study. That said, it is known that variations in hip shape exist between European and East Asian populations [1]. Future work, harnessing the resources of these large population studies with genetic data, could explore the incorporation of polygenic risk scores to assess whether their inclusion can enhance the performance of disease prediction models [37]. In addition, if more cohorts become available then superior trans-ancestry GWAS techniques become possible such as meta-regression of multi-ancestry genetic association (MR-MEGA) [38].

Although the present analysis focussed on genetic influences on individual HSMs and how they might affect risk of hip osteoarthritis, partitioning hip shape across combinations of HSMs known to be related to risk of hip osteoarthritis might yield stronger evidence of a causal relationship. For example, in equivalent studies based on knee shape, we generated a B-score reflecting the relationship between knee osteoarthritis and combinations of knee shape modes [39]. In future studies, we plan to examine the utility of this approach for studying the relationship between the risk of hip osteoarthritis and combinations of HSMs, including the role of genetic factors.

A limitation of this study is that UKB provides the majority of the participants (88%) and therefore the results are biased towards this population. Further work is justified to replicate these findings within a broader array of studies. A further limitation is the average age of the participants was ~ 60 years old, potentially leading to the coexistence of osteoarthritis in some individuals. However, the majority of participants did not exhibit any signs of radiological osteoarthritis (~90%), and all outline points, which were placed around the contour of the bones, were manually checked to make sure they did not encompass osteophytes [40]. Even if osteophytes were inadvertently included in our measures of hip shape, one would expect this to lead to false positive results from MR rather than the null associations found. Another limitation is the use of European reference panels for COJO and genetic correlation analyses that contained East Asian participants, which might result in biased estimates. That said, other ancestrally diverse GWAS meta-analyses have used European reference panel when conducting COJO with promising results [41]. Finally, there are known sex differences in hip shape and this study did not examine the X chromosome. Further work is needed to understand X chromosome based genetic pathways involved in the development of hip shape.

In conclusion, we present findings of a GWAS that investigated hip shape variation in the form of 10 HSMs. Despite finding considerable genetic overlap between hip shape and hip osteoarthritis, causal analyses suggest that rather than causing hip osteoarthritis, changes in hip shape could develop either in tandem with or in response to hip osteoarthritis. On the other hand, there was evidence that hip shape is a causal risk factor for hip fracture.

## Materials and methods

### Hip shape measurement

In both UKB and SC (cohort details in Supplementary Methods), eighty-five outline points were placed automatically around the proximal left femur to outline the femoral head, metaphysis, lesser and greater trochanters, and superior acetabulum. The outline points were placed inside osteophytes so these were not incorporated into the resulting shape measures as described previously [1, 21]. A statistical shape model was built from points fitted to all available images in UKB. Procrustes analyses removed size and rotational variation, producing a set of orthogonal principal components describing hip shape variation termed HSMs [21]. The UKB statistical shape model is now used as a reference model and in this study was applied to SC participants giving them scores for each HSM, which means the HSM scores are directly comparable between this study and previous studies [1, 21]. The first 10 HSMs were selected for analyses, as together they explained the majority (86.3%) of hip shape variance (Supplementary Fig. 16). HSMs and their associations with hip osteoarthritis have been described previously [21].

### Hip shape genetic analyses

The first 10 HSMs (standardized to mean = 0, standard deviation (SD) = 1) were used as outcomes in GWAS adjusted for age, sex, genotyping chip and the first 20 ancestry principal components in UKB, and age, sex and the first 10 ancestry principal components in SC (see Supplementary Methods for genetic imputation and quality control). Individuals with hip shape and genetic data available were included irrespective of osteoarthritis diagnosis. We tested SNP associations with each HSM assuming an additive allelic effect, in a linear mixed model implemented in BOLT-LMM v2.3.4 to account for cryptic population structure and relatedness in UKB. In SC we used fastGWA in GCTA v1.93.2 beta, a mixed linear model (MLM) approach to control for population stratification and relatedness. A centralized quality control of cohorts’ summary statistics was implemented in EasyQC (Supplementary Methods) [42]. Both cohorts used the hg19 build. Given the trans-ancestry nature of our GWAS, an additive random effects meta-analysis was performed with METAL for each HSM but fixed effect meta-analyses were also done as a sensitivity analysis [43, 44]. Following meta-analysis, SNPs with minor allele frequency (MAF) ≥ 0.01 in both cohorts were selected for further analyses.

### Fine mapping

Genome-wide complex trait analysis with conditional and joint genome-wide association analysis (GCTA-COJO) was used to identify statistically independent variants, which were mapped to the closest gene to define each locus so that duplicate loci could be identified across the 10 GWAS. A study-wide genome wide significance threshold was set at *P* < 5 × 10^−9^ to account for the 10 orthogonal HSMs examined. Previous GWAS hits for hip shape [9] and hip geometry [8] were looked up in this study to see if they replicated. The fine mapping methods are summarised in Supplementary Fig. 17 and the Supplementary Methods contain further details on GCTA-COJO, LDSC and fine mapping of loci. HSM GWAS signals were colocalised with expression quantitative trait loci (messenger RNA expression) taken from human joint tissue (synovium, and healthy and diseased cartilage) [45].

### Mendelian randomisation

Two-sample MR, which uses genetic proxies as instrumental variables, was conducted to estimate the causal effect of each HSM on hip osteoarthritis and hip fracture using the TwoSampleMR R package [46]. Conditionally independent variants from this study (*P* < 5×10^−8^) provided genetic instruments for HSMs. For outcomes, hip osteoarthritis data comprised a UKB GWAS of hospital diagnosed hip osteoarthritis, excluding participants included in the present HSM GWAS [19]. Hip fracture data comprised a previous GWAS meta-analysis [47] which includes UKB (the 5% sample overlap with the hip shape GWAS is unlikely to bias the MR results [48]). The IVW method was used as the primary analysis, where the causal estimate is obtained by combining the SNP-specific Wald ratios using a random-effects inverse-variance weighted meta-analysis. Strong evidence of a causal association was considered based on a Bonferroni adjusted *P* < 0.005 (as 10 HSMs tested). Sensitivity analyses to test the robustness of our estimates, included weighted median and mode, simple mode and Egger regression [12] (Supplementary Methods). Reverse MR was applied to understand the causal effect of a genetic predisposition of hip osteoarthritis and hip fracture on HSMs. Effect estimates represent a one SD increase for continuous exposures (i.e. HSM) and a doubling of odds for binary exposures (i.e. hip osteoarthritis and fracture).

## Supplementary Material

Supplementary_Figures_7_11_24_ddae169

Supp_Tables_16_8_24_ddae169

Supplementary_Methods_16_8_24_clean_ddae169

STROBE-MR-7_11_24_ddae169

## Data Availability

The HSM GWAS meta-analysis summary statistics will be uploaded to the GWAS catalog (https://www.ebi.ac.uk/gwas/). The individual level data from this study concerning UKB participants is available via their data showcase. Users must be registered with UK Biobank to access their resources (https://bbams.ndph.ox.ac.uk/ams/).
