# Supplementary material for: The genetic architecture of hip shape and its role in the development of hip osteoarthritis and fracture
Source: Hum Mol Genet. 2024 Nov 22;34(3):207–17. doi: 10.1093/hmg/ddae169 (PMC11792254; doi:10.1093/hmg/ddae169)
Supplement: Supplementary_Figures_7_11_24_ddae169 [file supplementary_figures_7_11_24_ddae169.docx]

Supplementary Figures


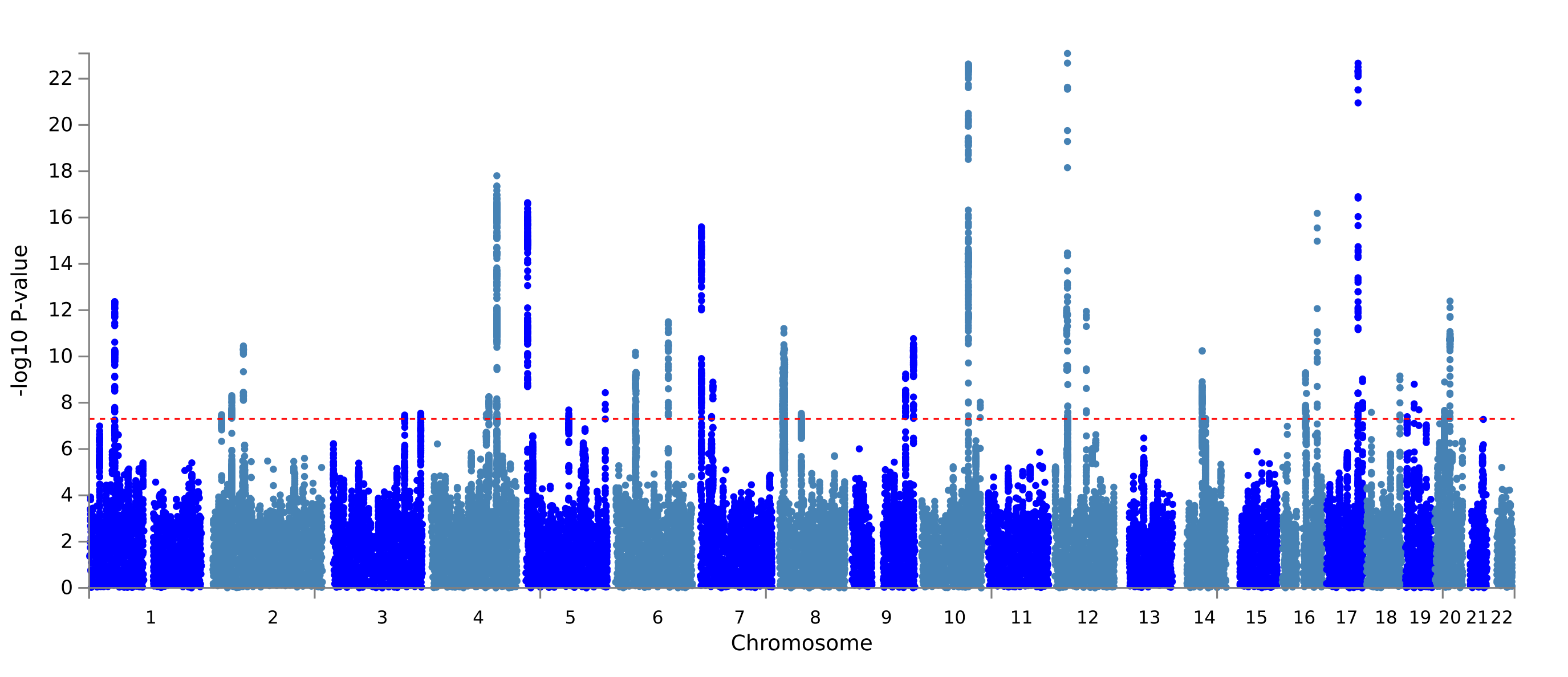

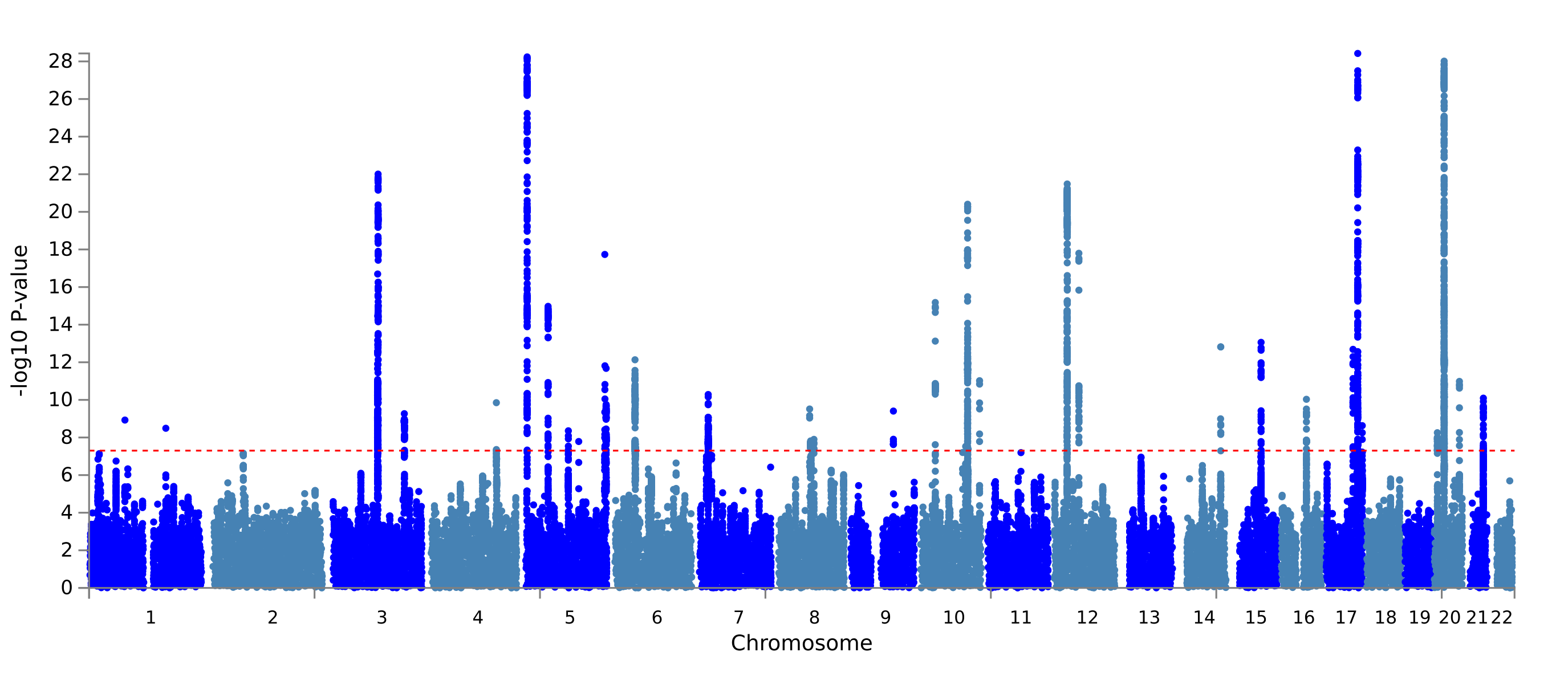

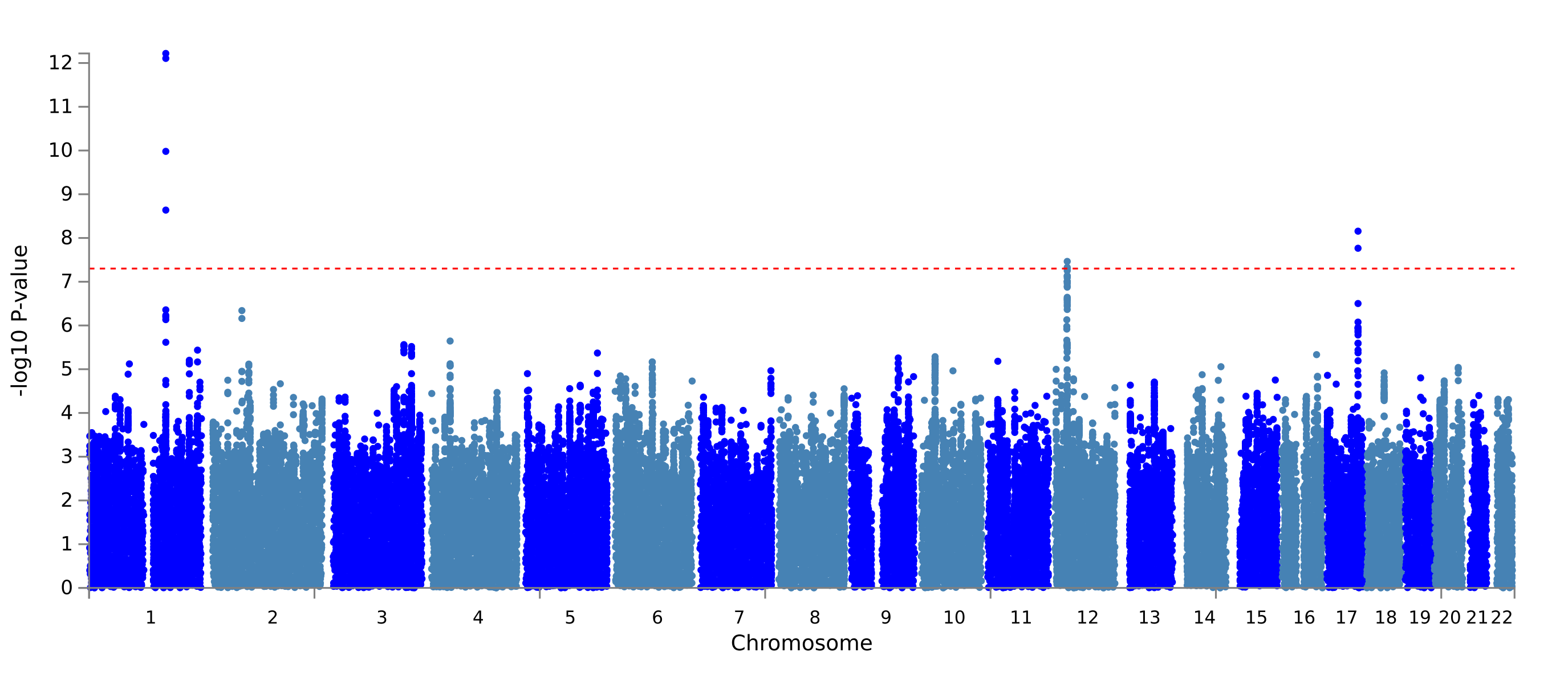

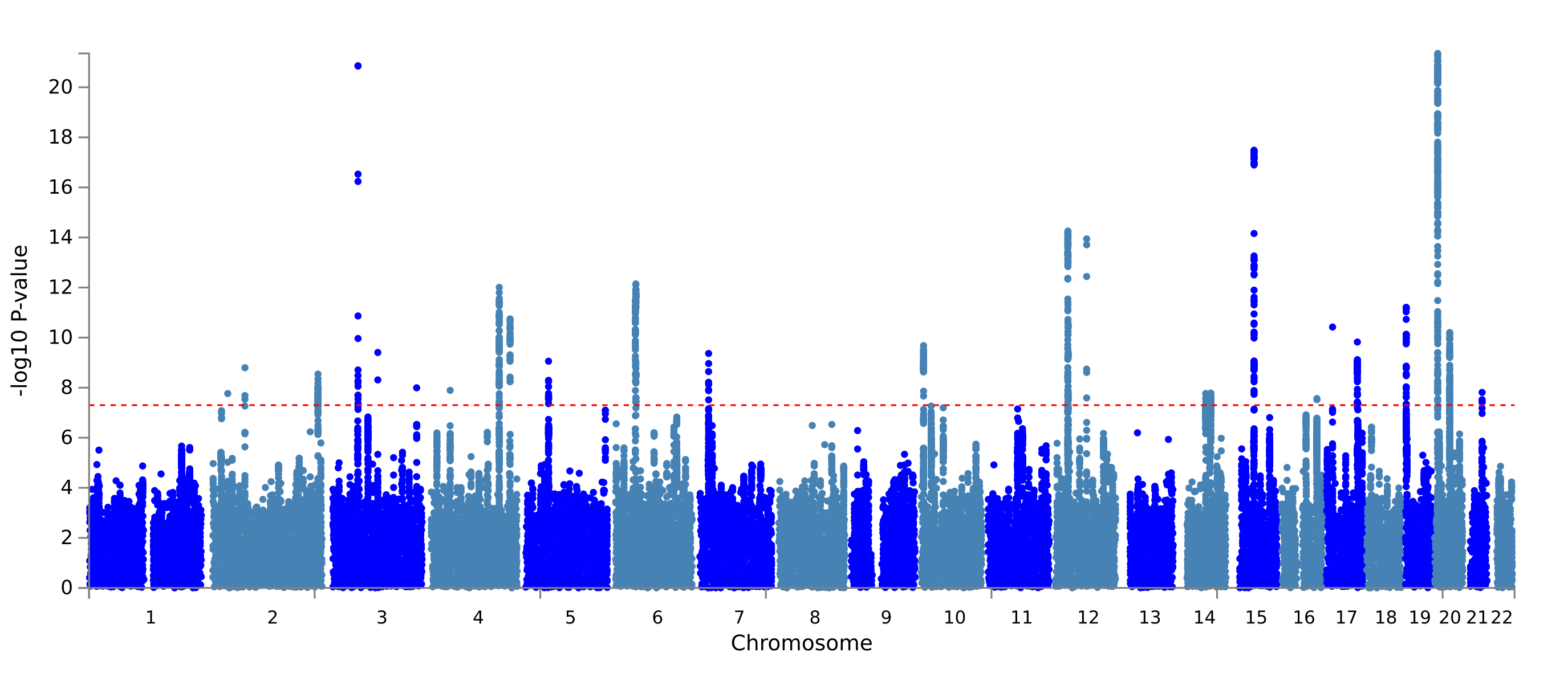

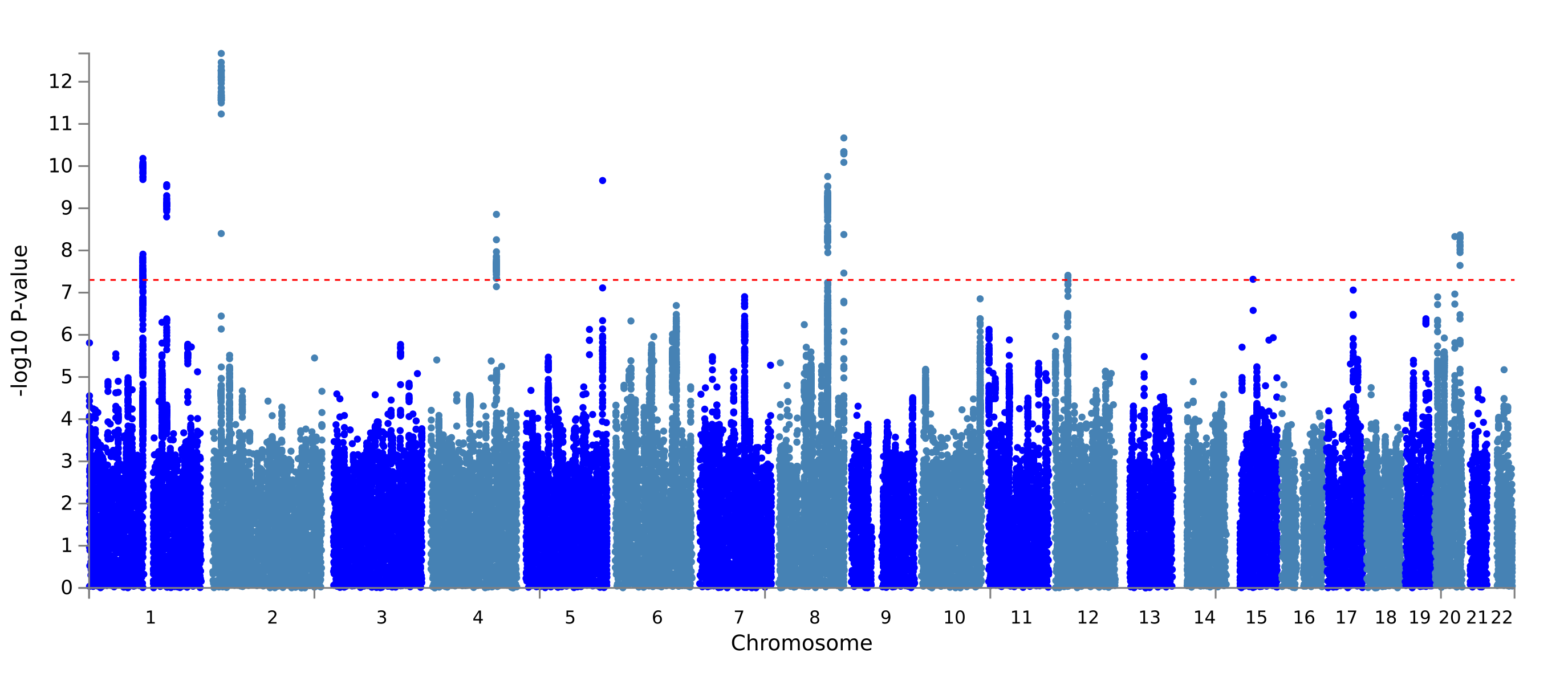

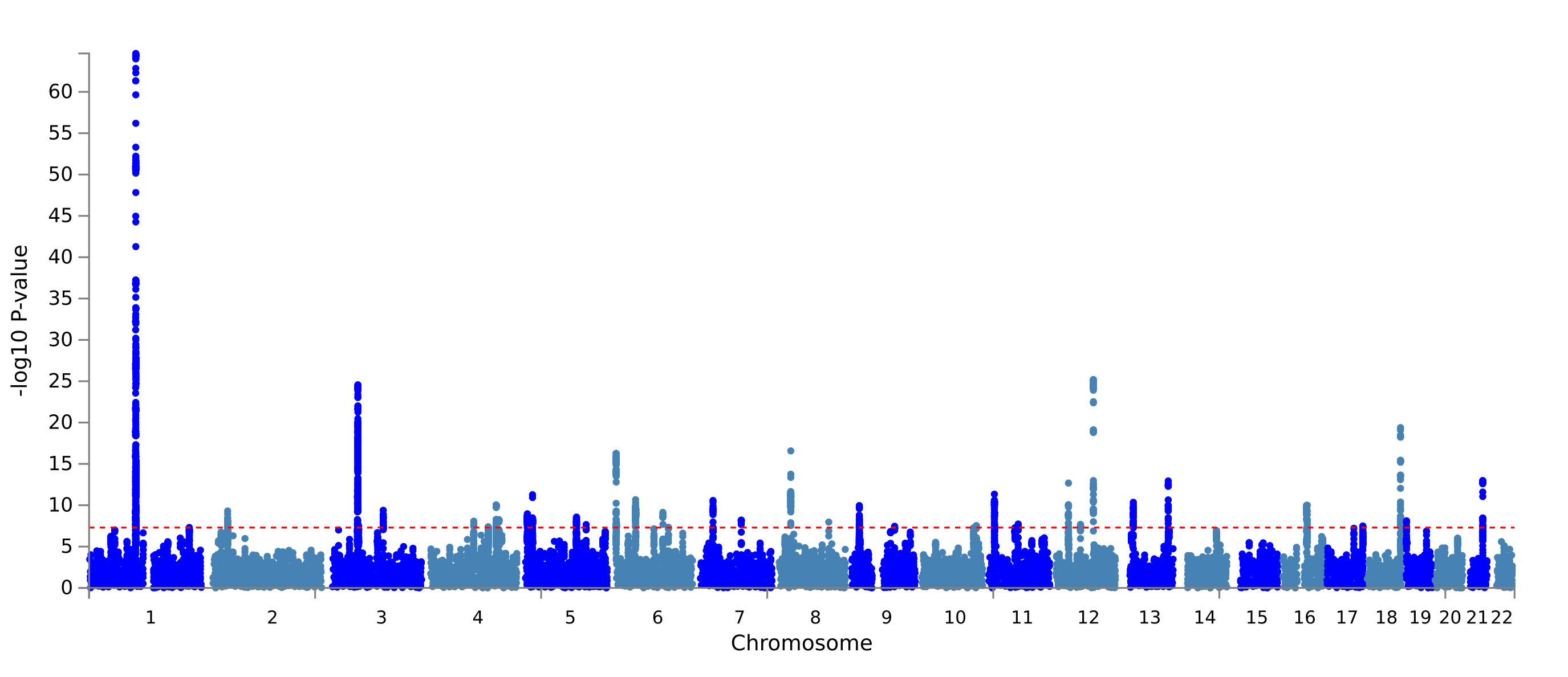

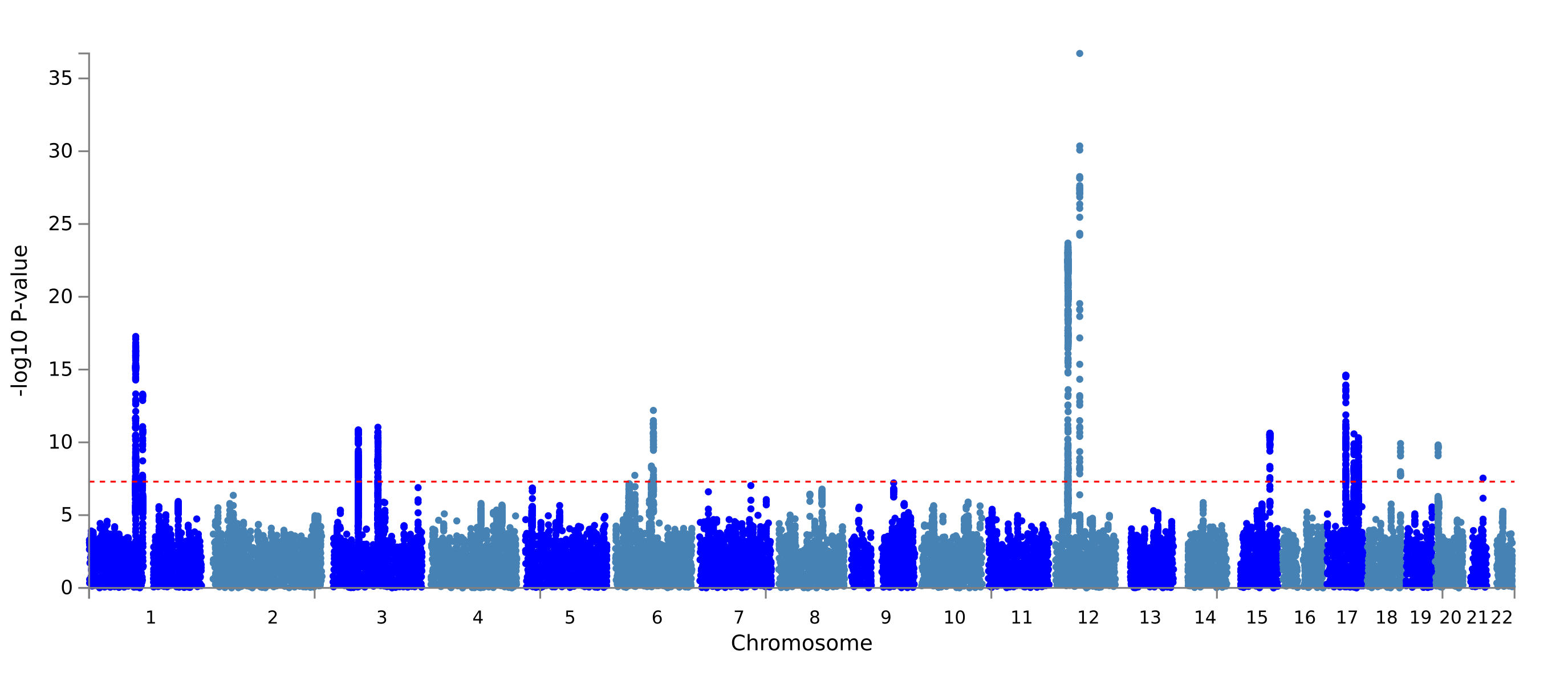

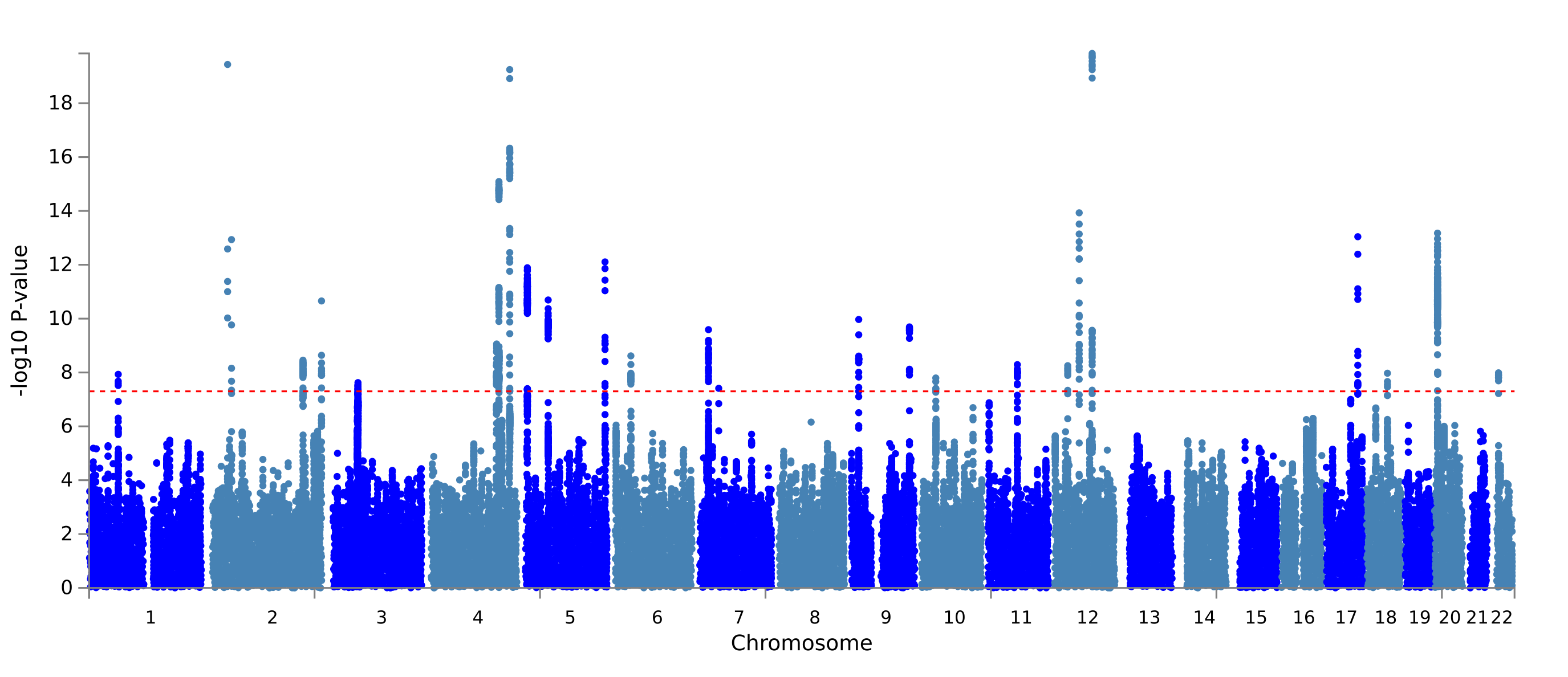


Supplementary Figure 1. A Manhattan plot for hip shape mode 1.


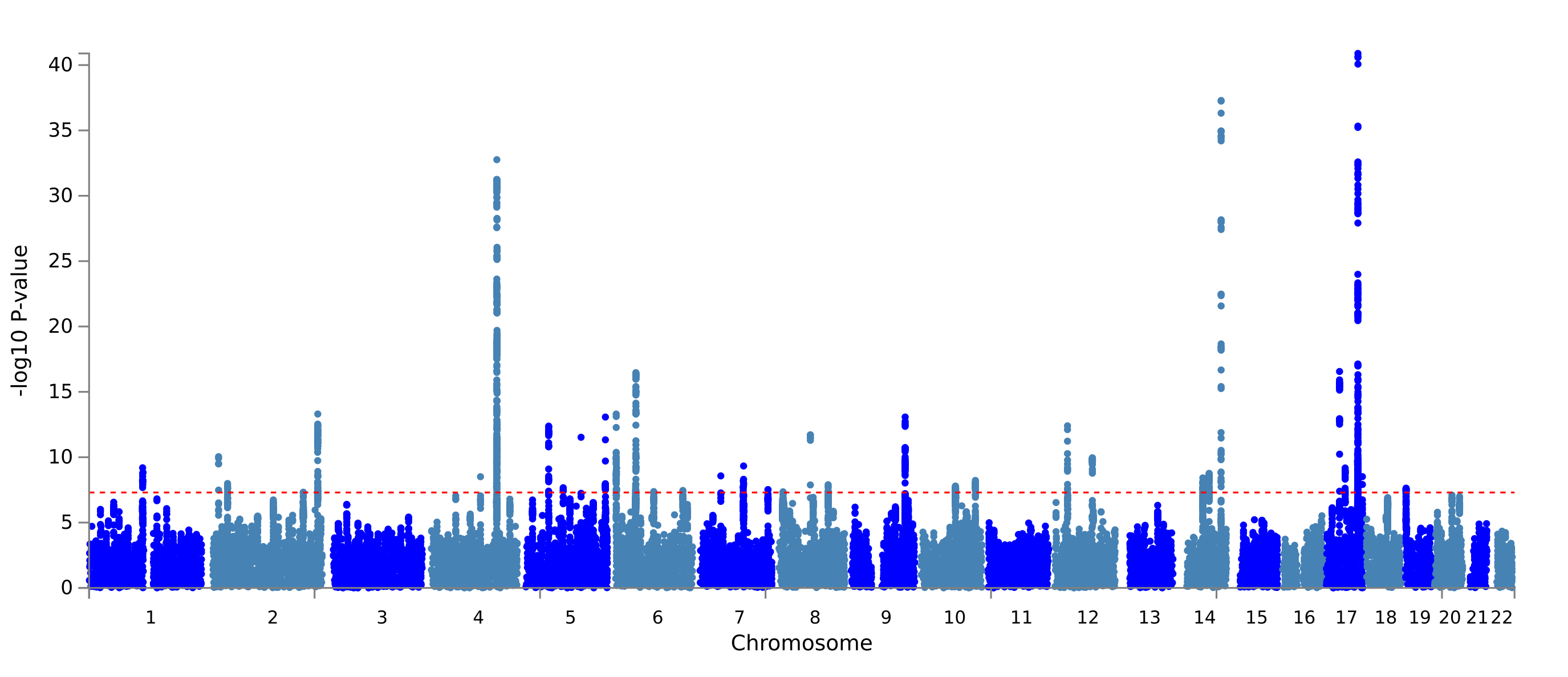


Supplementary Figure 2. A Manhattan plot for hip shape mode 2.


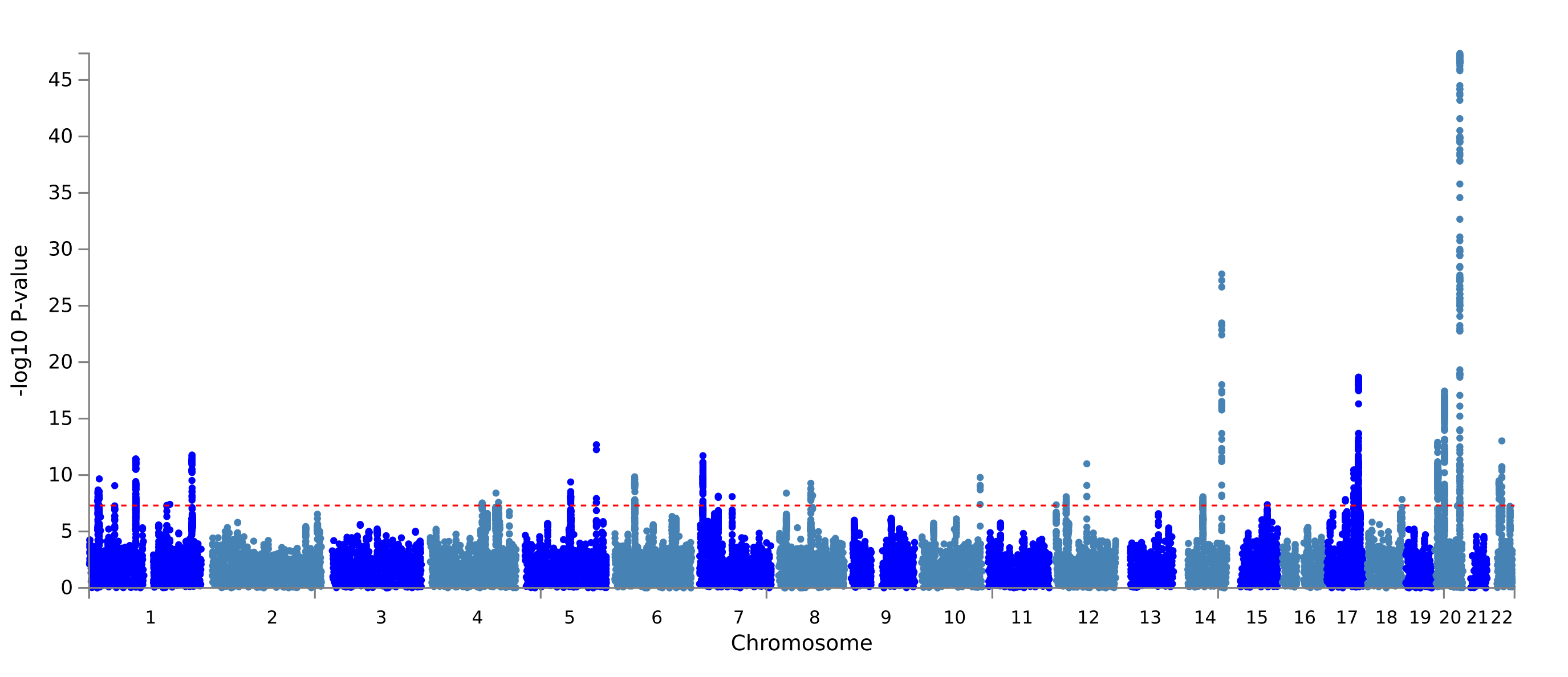


Supplementary Figure 3. A Manhattan plot for hip shape mode 3.


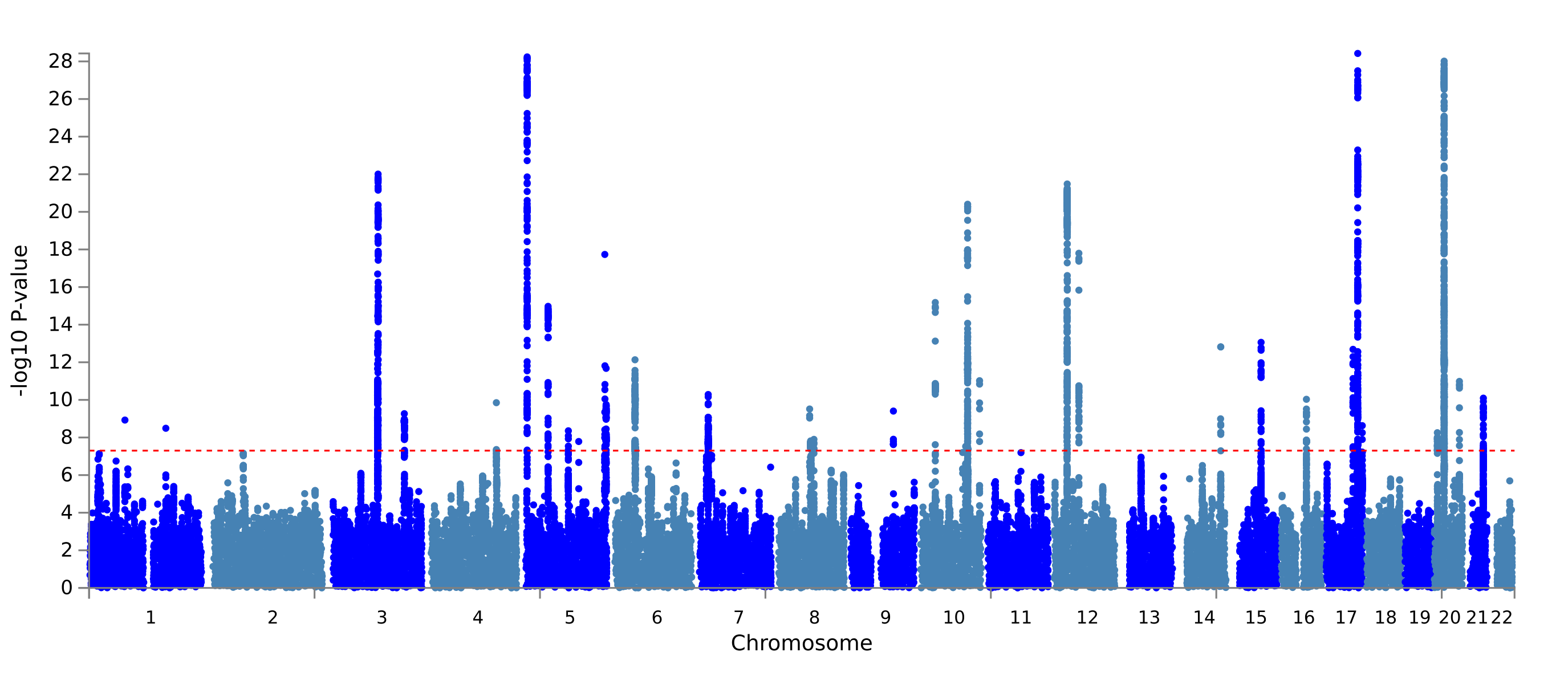


Supplementary Figure 4. A Manhattan plot for hip shape mode 4.


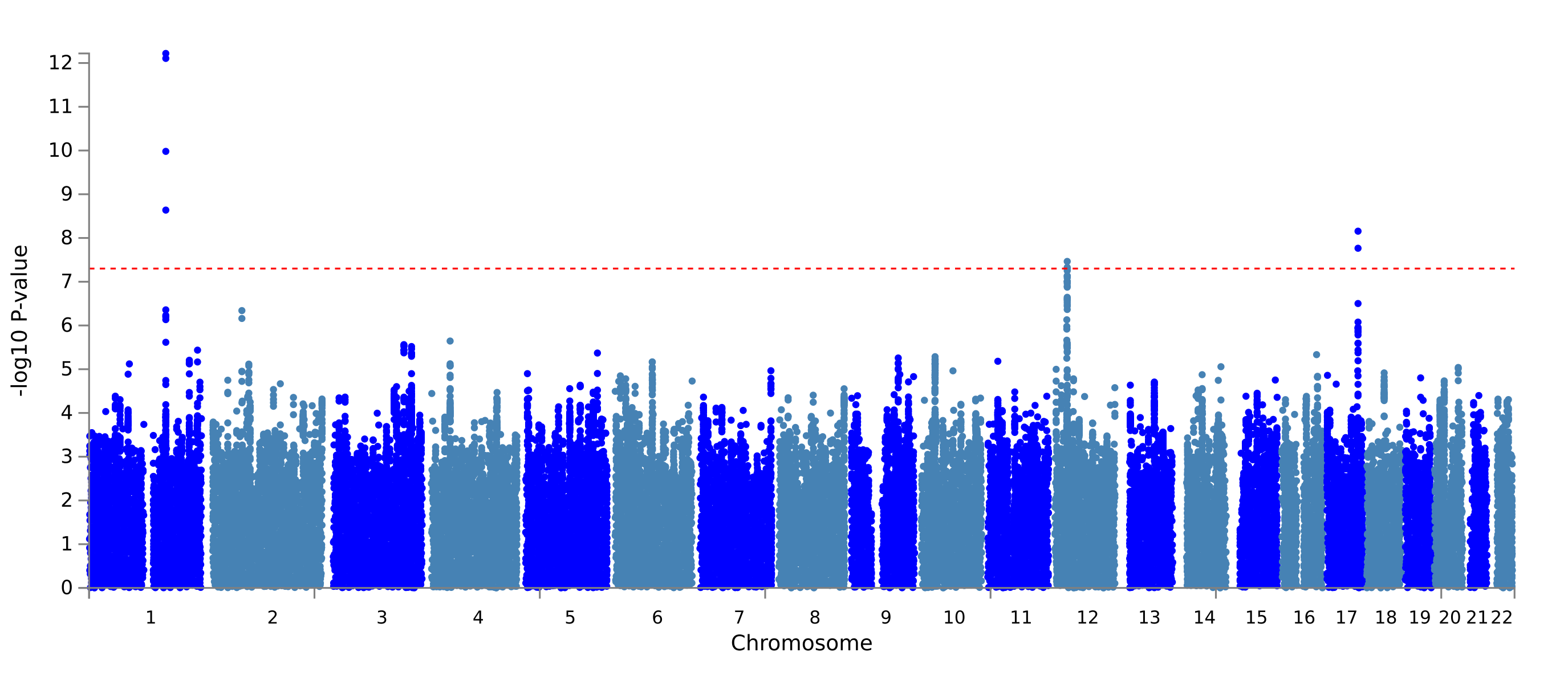


Supplementary Figure 5. A Manhattan plot for hip shape mode 5.


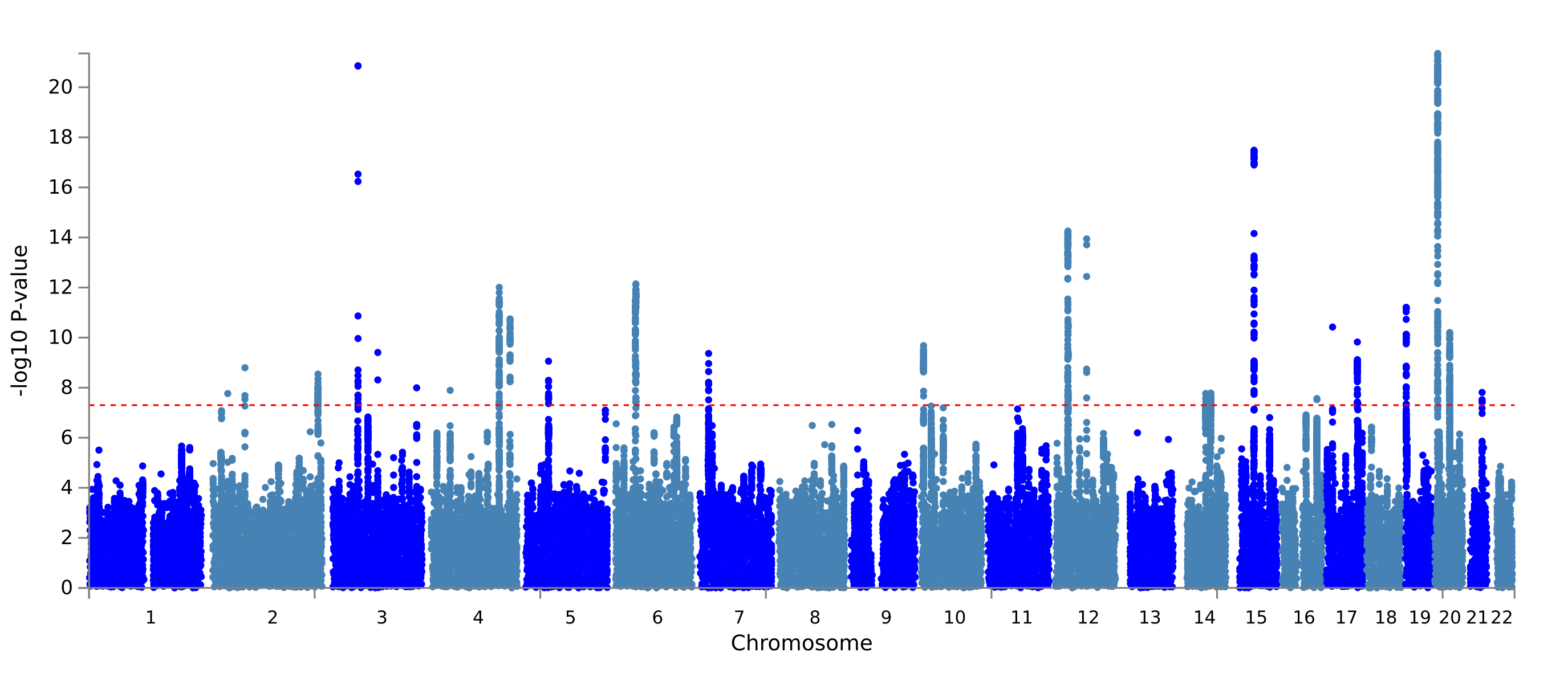


Supplementary Figure 6. A Manhattan plot for hip shape mode 6.


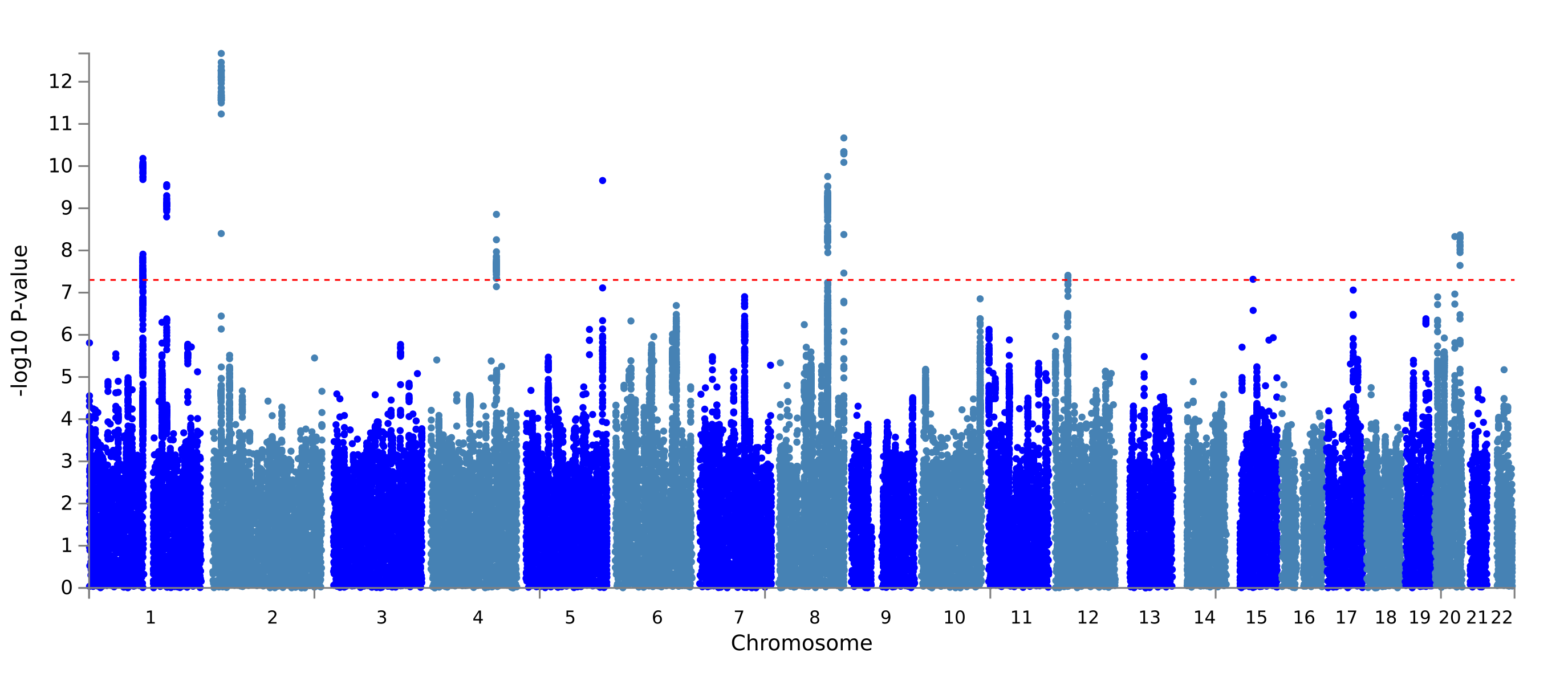


Supplementary Figure 7. A Manhattan plot for hip shape mode 7.


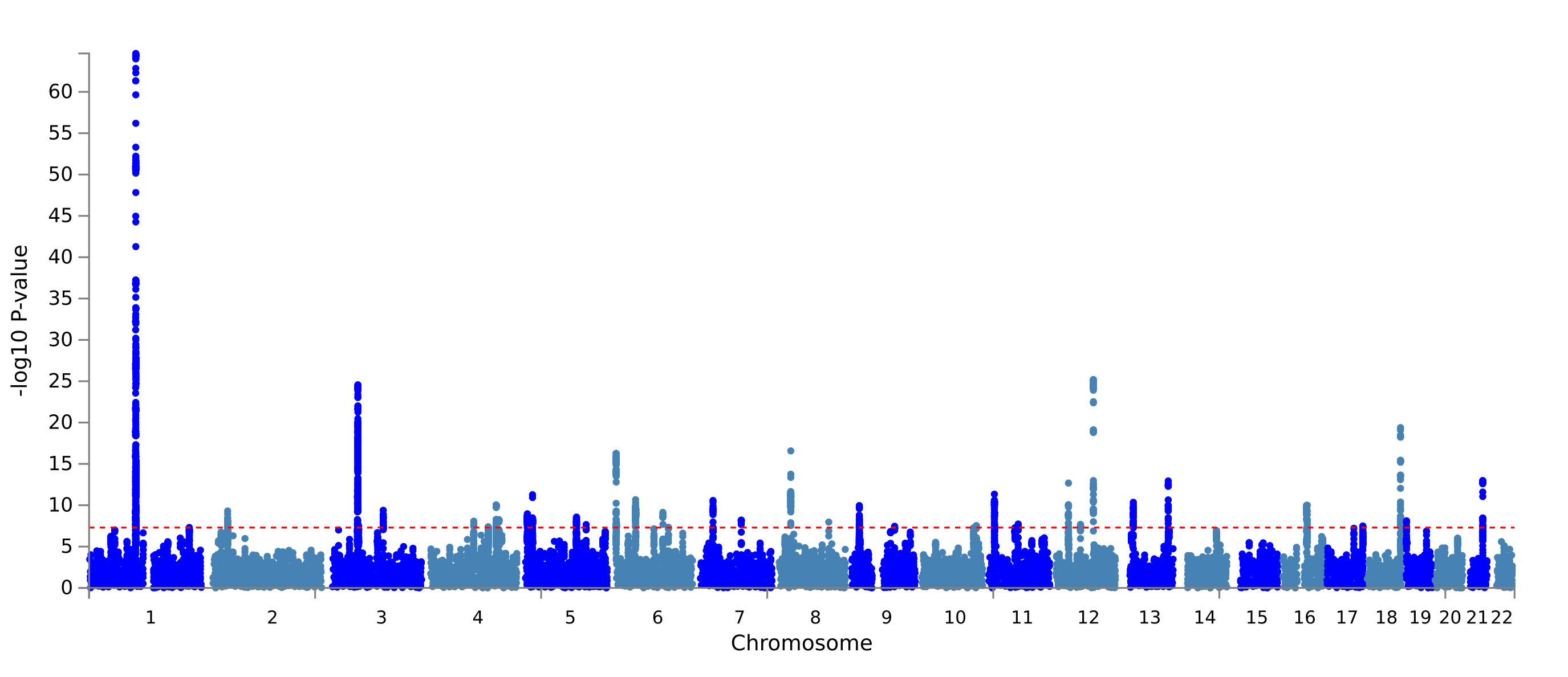


Supplementary Figure 8. A Manhattan plot for hip shape mode 8.


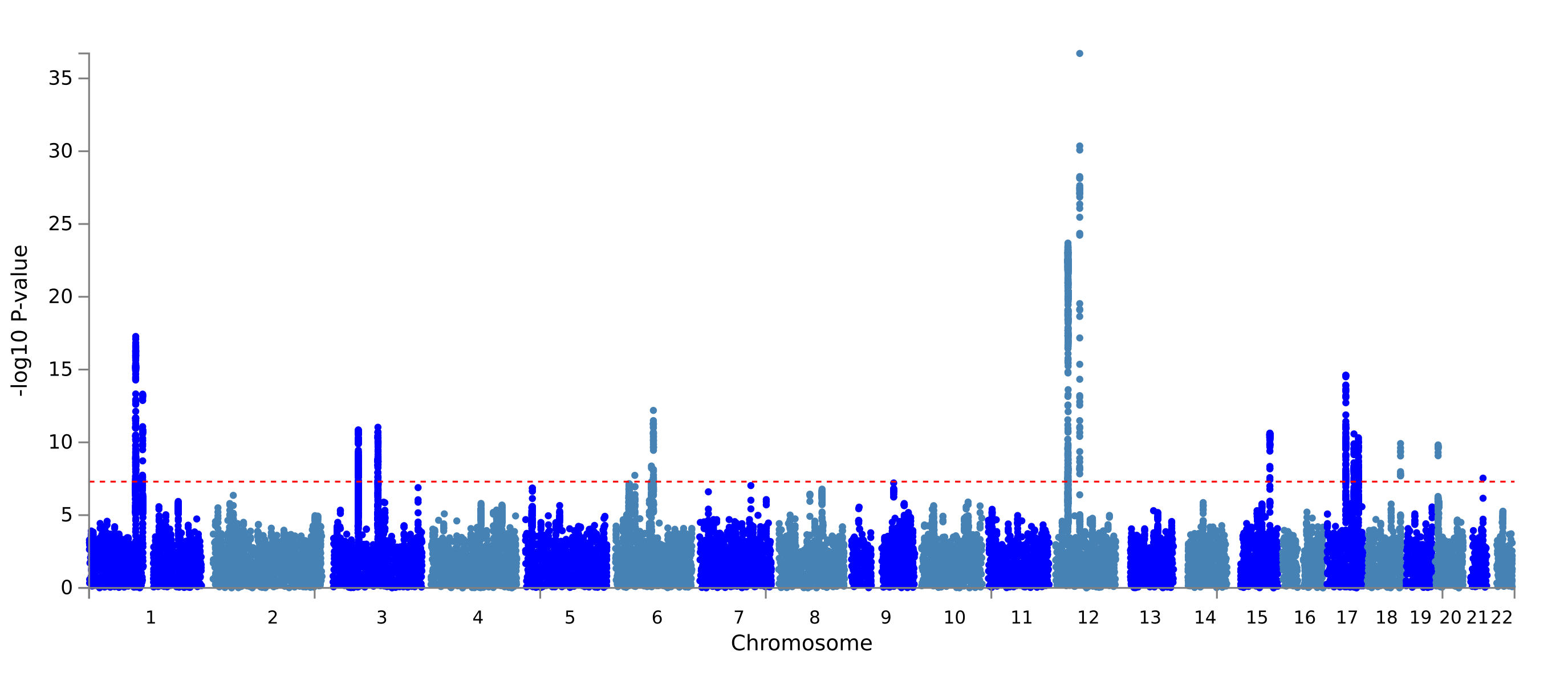


Supplementary Figure 9. A Manhattan plot for hip shape mode 9.


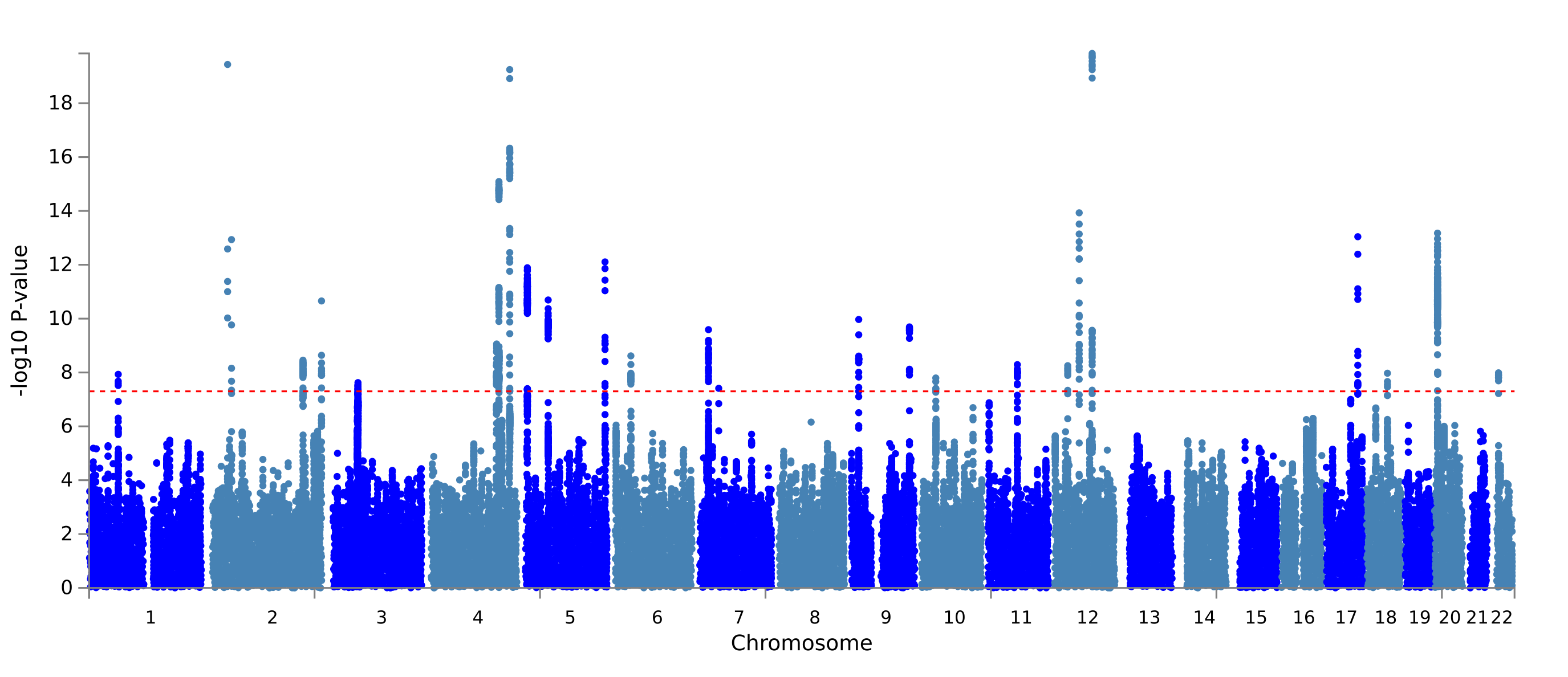


Supplementary Figure 10. A Manhattan plot for hip shape mode 10.

Supplementary Figure 11.


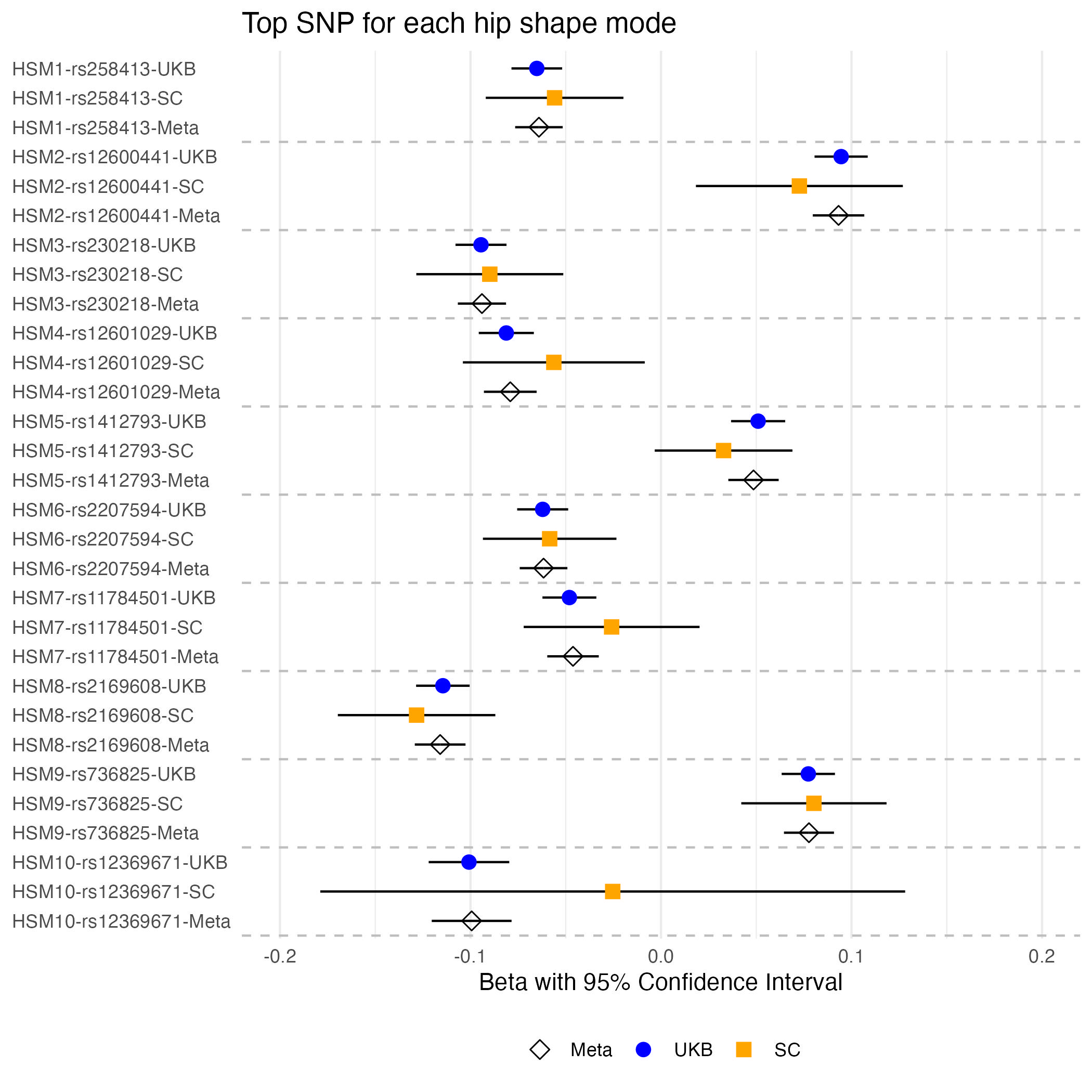


The SNP with the smallest p-value per hip shape mode was selected for this figure to illustrate the differences in effect estimates between the cohorts. This shows UKB exerts the greatest influence on meta-analysis effect estimates, due to its larger sample siz. UKB – UK Biobank, SC – Shanghai Changfeng, Meta – random-forest meta-analysis


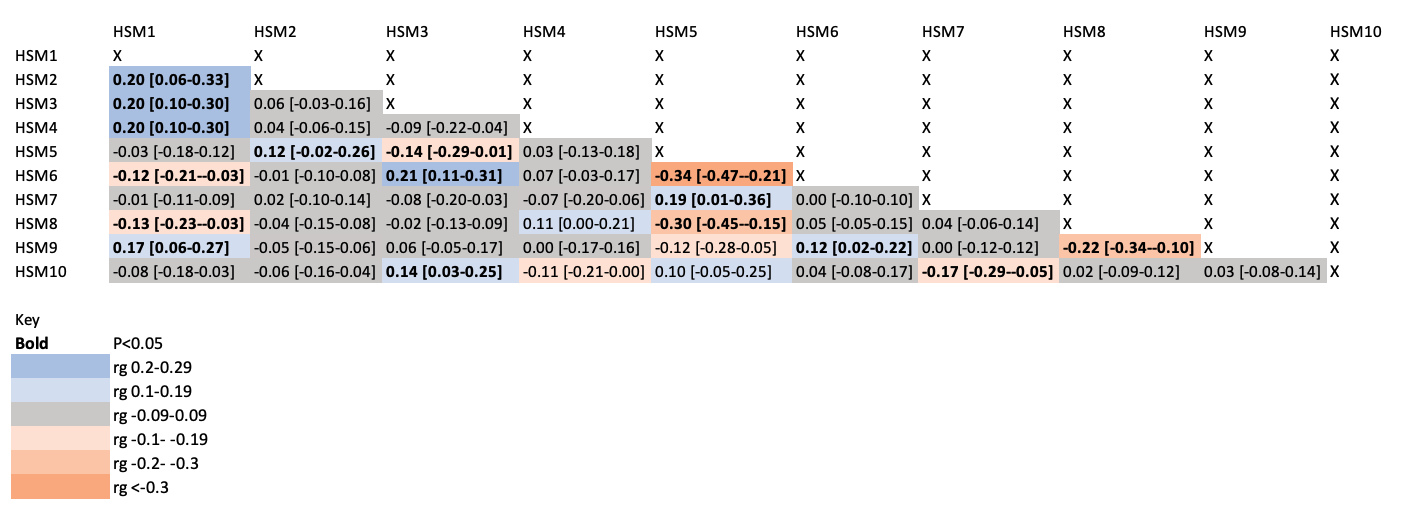


Supplementary Figure 12. Genetic correlations between hip shape modes from the random effects meta-analysis. 95% confidence interval within square brackets. HSM – hip shape mode. Rg – correlation coefficient.


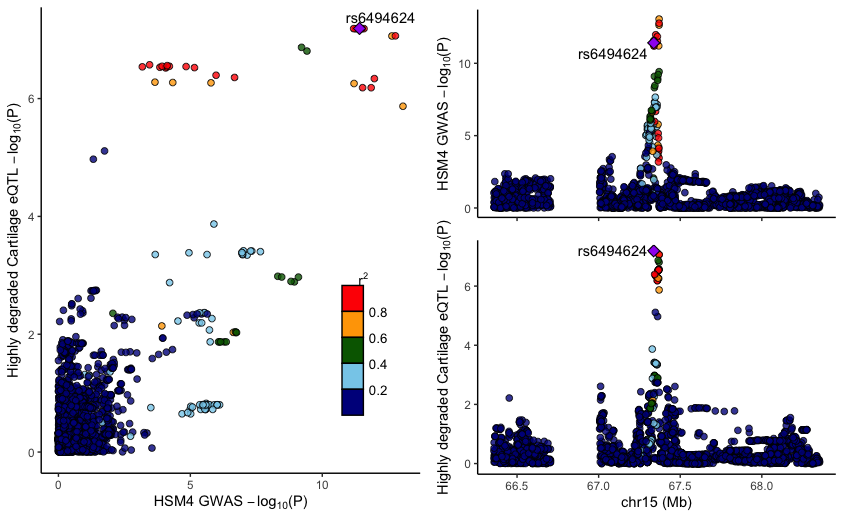


Supplementary Figure 13. Colocalisation plot between *SMAD3* expression quantitative trait loci signal and HSM4 GWAS in highly degraded human cartilage.


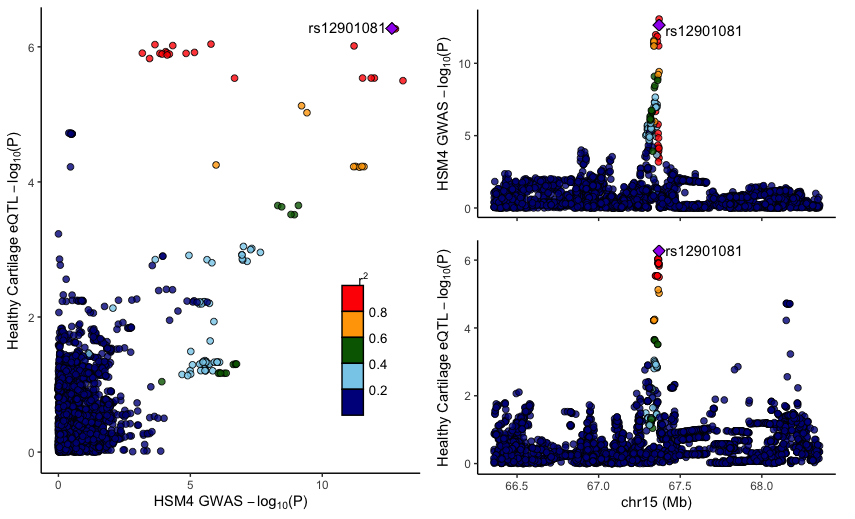


Supplementary Figure 14. Colocalisation plot between *SMAD3* expression quantitative trait loci signal and HSM4 GWAS in healthy human cartilage.


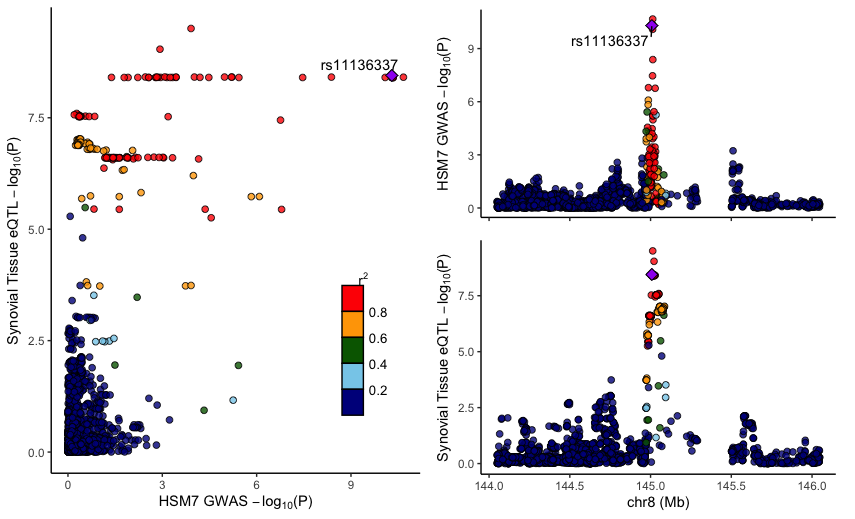


Supplementary Figure 15. Colocalisation plot between *PLEC* expression quantitative trait loci signal and HSM7 GWAS in healthy human cartilage.

Supplementary Figure 16. Scree plot displaying shape variance captured by each hip shape mode


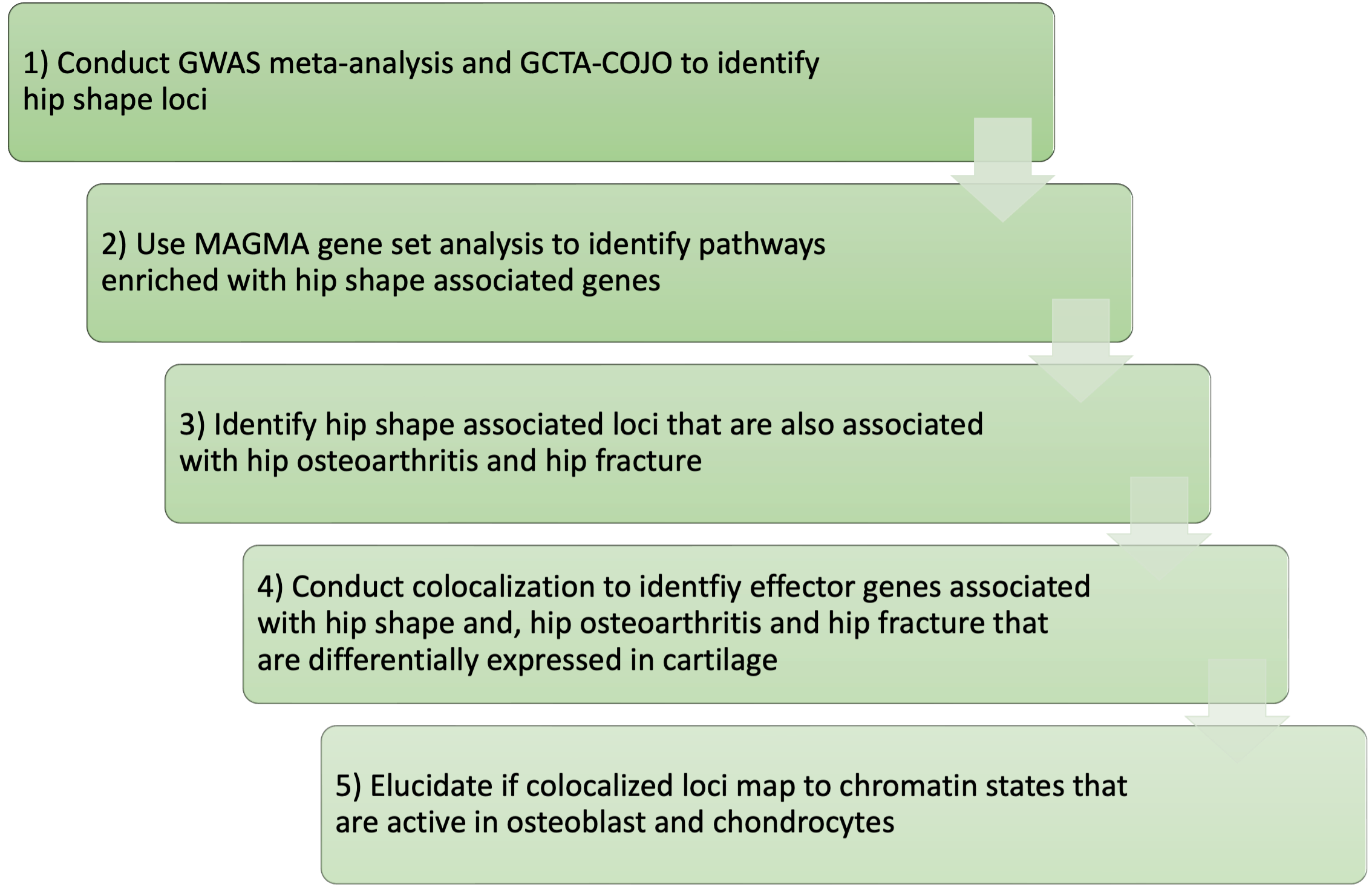


Supplementary Figure 17. Methodological process for genetic association fine-mapping
