# Supplementary material for: The genetic architecture of hip shape and its role in the development of hip osteoarthritis and fracture
Source: Hum Mol Genet. 2024 Nov 22;34(3):207–17. doi: 10.1093/hmg/ddae169 (PMC11792254; doi:10.1093/hmg/ddae169)
Supplement: Supplementary_Methods_16_8_24_clean_ddae169 [file supplementary_methods_16_8_24_clean_ddae169.docx]

## Supplementary Methods

### Study population

#### UK Biobank

UKB is a prospective cohort study which recruited 500,000 adults from the United Kingdom, aged between 40 and 69 years at a baseline visit which took place between 2006-2010 (1). The participants have undergone comprehensive genetic and physical phenotyping (http://biobank.ctsu.ox.ac.uk/crystal/). The study is overseen by the Ethics Advisory Committee and received approval from the National Information Governance Board for Health and Social Care and Northwest Multi-Centre Research Ethics Committee (11/NW/0382), all participants provided informed consent for this study. As part of the UKB extended imaging study, which commenced in 2014, a number of imaging modalities, including dual-energy X-ray absorptiometry (DXA) scans (iDXA, GE-Lunar) of the hip are being collected (2).

#### Shanghai Changfeng

Between 2009 to 2012, a total of 6,595 Chinese participants (over 45 years old) from SC Community were recruited by the SC study (3). Left hip DXAs (iDXA, GE-Lunar) and genetic information were collected in 5,310 participants which met imaging and genotyping quality control. Ethics approval was granted by the ethics committee of Zhongshan Hospital affiliated to Fudan University and written informed consent was provided by all participants.

### Genotyping and QC

#### UKB

Genotyping, imputation and quality control (QC) were performed by UKB as previously described (1). Samples were genotyped using two genotyping arrays; Applied Biosystems UK BiLEVE Axiom Array by Affymetrix (49,950 participants) and Applied Biosystems UK Biobank Axiom Array (438,427 participants). Data were imputed using the HRC reference panel, and the merged UK10K and 1000 Genomes phase 3 reference panels in IMPUTE4. A subset of individuals with European ancestry used for the UKB HSM GWAS, which accounted for 95% (38,175/40,311) of participants with available hip shape data. This was to reduce the possibility of spurious results from small numbers of ancestrally diverse individuals in UK Biobank. Ancestry assignment of UKB participants was performed as follows: the UKB sample was projected onto the first 20 principal components estimated from the 1000 Genomes Phase 3 (1000G) project (where ancestry was known) using GCTA version 1.93.2. Projections used a curated set of 38,512 LD-pruned HapMap 3 Release 3 (HM3) bi-allelic SNPs that were shared between the 1000G and UKB genotyped datasets (i.e. MAF > 1%, minor allele count > 5, genotyping call rate > 95%, Hardy-Weinberg P > 1x10^-6^, and regions of extensive LD removed). Uniform Manifold Approximation and Projection for Dimension Reduction (UMAP) was used in conjunction with the first 20 principal components to cluster 486,445 individuals using the following parameters: min_dist=0.0001, n_components=3, n_neighbors=45, random_state=10293082. UKB participants that clustered together with the 1000G European sub-populations were manually identified by visual inspection (N=461,920) and used for downstream genetic analyses.

#### SC

Genotyping, imputation and quality control (QC) were performed by SC as previously described (4). Genomic DNA of the participants were extracted from peripheral blood leukocytes using QIAGEN (Hilden, Germany) QIAamp DNA Mini Blood Kit and genotyped with Illumina (San Diego, CA, USA) Infinium BeadChip genotyping array (707,180 markers). Quality control was carried out using PLINK v1.90b4 (i.e. MAF > 1%, Genotype missingness < 2%, Hardy-Weinberg p >1e-5). Samples that had a sex discrepancy between genotypic and reported sex, or had genotype hard call rate <95%, or deviated from the expected inbreeding coefficient (-0.2<F<0.2) or were duplicated samples were excluded. We performed pre-phasing and phasing using SHAPEIT v2r790 and imputation with IMPUTE2 v2.3.1 with the 1000 Genomes (1000G) phase 3 data as the reference. Quality control was carried out again and 8,393,320 variants with call rate >98%, Hardy-Weinberg p >1e-5, MAF >1%, and INFO score >0.8 were included in the final analysis. All genomic positions were in reference to hg19/build 37.

### Easy QC

Missing data, mono allelic SNPs, implausible values (P > 1, infinite SE, beta >10, EAF>1), variants with poor imputation quality (INFO <0.4) and minor allele frequency ≤0.01, and duplicates were removed prior to meta-analysis. Allele coding was harmonized across both cohorts (A/T/C/G or I/D) and allele frequency checked against HRC 1.1 imputed reference (<http://www.haplotype-reference-consortium.org>) for UKB and checked against 1000Genome phase 3 version 5 East Asian reference for SC (<https://www.uni-regensburg.de/medizin/epidemiologie-praeventivmedizin/genetische-epidemiologie/software/>) to account for the ancestral differences between the populations. Scatter plots were inspected to identify problems with beta estimates, standard errors and *P* values.

### Identifying conditionally independent variants

To identify statistically independent variants, we applied approximate conditional and joint genome-wide association analysis (GCTA-COJO) (5). SNPs with high collinearity (multiple regression R^2^ > 0.9) were excluded from the analysis. A reference sample of unrelated individuals of European origin, randomly selected from UKB was used to model patterns of linkage disequilibrium (LD) between variants. Conditionally independent variants reaching GWAS significance were annotated to the physically closest gene using BEDTools (6) v2.3.0 and the Hg19 Gene range list available from https://useast.ensembl.org/info/data/biomart/index.html

### SNP heritability and genetic correlation

Linkage disequilibrium (LD) score regression (LDSC) v1.0.1 was used to estimate SNP heritability, and the genetic correlation between each HSM and hip osteoarthritis (7) and hip fracture (8). A European reference panel (1000 Genome) was used for the meta-analysis and UKB whilst an East Asian Reference panel (1000 Genome) was used for SC. (9). In addition, the genetic correlations between HSM_UKB_ and HSM_SC_ were examined using LDSC with a European reference panel. These analyses were limited to HapMap3 SNPs (excluding major histocompatibility regions) (9).

### Fine mapping

To understand if any variants associated with hip shape were associated with hip osteoarthritis and fracture, we conducted a look up of all conditionally independent SNPs in previous GWAS of both conditions (7, 8). We also examined estimated bone mineral density and other osteoarthritis traits including: osteoarthritis at all sites, osteoarthritis at the knee and/or hip and total hip replacement (7, 10). A less-stringent Bonferroni adjusted P-value threshold (P<2.5x10^-4^) was used to account for 203 SNPs tested (0.05/203 = 2.5x10^-4^) allowing for the identification of suggestive loci which did not meet genome-wide significance in the original study. For those hip shape SNPs which were associated with either hip osteoarthritis or fracture, we used the coloc R package, to compare 100kb regions on either side of the lead hip shape SNP with the relevant disease GWAS to look for shared GWAS signals (11). In addition, generalised gene-set analysis of GWAS data (MAGMA v1.08) (12) was implemented in Functional Mapping and annotation of GWAS (FUMA) tool (13). Briefly, SNPs were mapped to the protein coding genes using default settings (SNP-wise (mean) model for gene test) and gene-set analysis was performed using 18824 gene sets obtained from MsigDB v5.2. To identify which cis-genes share the same causal variants, we used colocalisation to look at eQTL data 1Mb either side of the hip shape variant, obtained from human highly degraded (diseased) and less degraded (healthy) cartilage, and synovial tissue (14). We considered a SNP to colocalise with an eQTL if the fourth posterior probability (PP) was >80% and suggestive of colocalising if the PP was >60% but <80%. In addition, regulatory elements of non-coding human genome were identified using RegulomeDB (15), the deleterious potential of the variant by combined annotation dependent depletion (CADD) (16) and its 15-core chromatin state within chondrocytes and osteoblasts prediction by ChromoHMM (17). CADD and ChromoHMM estimates were obtained through FUMA (13).

### Mendelian randomisation

Sensitivity analyses were used to test the conclusion of our Mendelian randomisation analyses. These analyses relax the assumption of no horizontal pleiotropy, assuming uncorrelated pleiotropy (MR-Egger), or relax the assumption about the number of invalid instruments (weighted median, simple mode and weighted mode).

1. Bycroft C, Freeman C, Petkova D, Band G, Elliott LT, Sharp K, et al. The UK Biobank resource with deep phenotyping and genomic data. Nature. 2018;562(7726):203-9.

2. Littlejohns TJ, Holliday J, Gibson LM, Garratt S, Oesingmann N, Alfaro-Almagro F, et al. The UK Biobank imaging enhancement of 100,000 participants: rationale, data collection, management and future directions. Nature communications. 2020;11(1):2624.

3. Gao X, Hofman A, Hu Y, Lin H, Zhu C, Jeekel J, et al. The Shanghai Changfeng Study: a community-based prospective cohort study of chronic diseases among middle-aged and elderly: objectives and design. Eur J Epidemiol. 2010;25(12):885-93.

4. Zeng H, Ge J, Xu W, Ma H, Chen L, Xia M, et al. Twelve Loci Associated With Bone Density in Middle-aged and Elderly Chinese: The Shanghai Changfeng Study. J Clin Endocrinol Metab. 2023;108(2):295-305.

5. Yang J, Ferreira T, Morris AP, Medland SE, Genetic Investigation of ATC, Replication DIG, et al. Conditional and joint multiple-SNP analysis of GWAS summary statistics identifies additional variants influencing complex traits. Nat Genet. 2012;44(4):369-75, S1-3.

6. Quinlan AR, Hall IM. BEDTools: a flexible suite of utilities for comparing genomic features. Bioinformatics. 2010;26(6):841-2.

7. Boer CG, Hatzikotoulas K, Southam L, Stefansdottir L, Zhang Y, Coutinho de Almeida R, et al. Deciphering osteoarthritis genetics across 826,690 individuals from 9 populations. Cell. 2021;184(18):4784-818 e17.

8. Nethander M, Coward E, Reimann E, Grahnemo L, Gabrielsen ME, Wibom C, et al. Assessment of the genetic and clinical determinants of hip fracture risk: Genome-wide association and Mendelian randomization study. Cell Rep Med. 2022;3(10):100776.

9. Bulik-Sullivan B, Finucane HK, Anttila V, Gusev A, Day FR, Loh PR, et al. An atlas of genetic correlations across human diseases and traits. Nat Genet. 2015;47(11):1236-41.

10. Morris JA, Kemp JP, Youlten SE, Laurent L, Logan JG, Chai R, et al. An Atlas of Human and Murine Genetic Influences on Osteoporosis. 2018.

11. Giambartolomei C, Vukcevic D, Schadt EE, Franke L, Hingorani AD, Wallace C, et al. Bayesian test for colocalisation between pairs of genetic association studies using summary statistics. PLoS Genet. 2014;10(5):e1004383.

12. de Leeuw CA, Mooij JM, Heskes T, Posthuma D. MAGMA: generalized gene-set analysis of GWAS data. PLoS Comput Biol. 2015;11(4):e1004219.

13. Watanabe K, Taskesen E, van Bochoven A, Posthuma D. Functional mapping and annotation of genetic associations with FUMA. Nat Commun. 2017;8(1):1826.

14. Steinberg J, Southam L, Roumeliotis TI, Clark MJ, Jayasuriya RL, Swift D, et al. A molecular quantitative trait locus map for osteoarthritis. Nat Commun. 2021;12(1):1309.

15. Boyle AP, Hong EL, Hariharan M, Cheng Y, Schaub MA, Kasowski M, et al. Annotation of functional variation in personal genomes using RegulomeDB. Genome Res. 2012;22(9):1790-7.

16. Kircher M, Witten DM, Jain P, O'Roak BJ, Cooper GM, Shendure J. A general framework for estimating the relative pathogenicity of human genetic variants. Nat Genet. 2014;46(3):310-5.

17. Ernst J, Kellis M. ChromHMM: automating chromatin-state discovery and characterization. Nat Methods. 2012;9(3):215-6.
