# Supplementary material for: The genetic architecture of hip shape and its role in the development of hip osteoarthritis and fracture
Source: Hum Mol Genet. 2024 Nov 22;34(3):207–17. doi: 10.1093/hmg/ddae169 (PMC11792254; doi:10.1093/hmg/ddae169)
Supplement: STROBE-MR-7_11_24_ddae169 [file strobe-mr-7_11_24_ddae169.docx]

**STROBE-MR checklist of recommended items to address in reports of Mendelian randomization studies**^1^ ^2^

| **Item No.** | **Section** | **Checklist item** | **Page No.** | **Relevant text from manuscript** |
| --- | --- | --- | --- | --- |
| 1 | **TITLE and ABSTRACT** | Indicate Mendelian randomization (MR) as the study’s design in the title and/or the abstract if that is a main purpose of the study | 2 | Contained in the abstract: “Two-sample Mendelian randomisation (MR) was used to estimate causal effects between HSM and hip osteoarthritis using hip fracture as a positive control.” |
|  | **INTRODUCTION** |  |  |  |
| 2 | **Background** | Explain the scientific background and rationale for the reported study. What is the exposure? Is a potential causal relationship between exposure and outcome plausible? Justify why MR is a helpful method to address the study question | 3 | “The observational studies showing an association between hip shape and hip osteoarthritis are prone to confounding (13, 14). For example, it has been reported that young athletes are at increased risk of cam morphology (4, 15, 16). However, this observed association might result from confounding due to excess physical activity causing hip osteoarthritis, rather than cam morphology. Differentiating between a causal and confounded association is important as surgical correction of hip shape has been proposed to prevent or delay the onset of hip osteoarthritis and trials are ongoing (17, 18); it has been difficult to test this hypothesis through randomised control trials due to the slow onset of hip osteoarthritis (13). “ |
| 3 | **Objectives** | State specific objectives clearly, including pre-specified causal hypotheses (if any). State that MR is a method that, under specific assumptions, intends to estimate causal effects | 3 | “GWAS can also provide genetic instruments for traits that be used in Mendelian randomisation (MR) to estimate causal effects. This method is less susceptible to confounding and has been likened to a natural randomised control study that does not require years of follow up (10-12).” |
|  | **METHODS** |  |  |  |
| 4 | **Study design and data sources** | Present key elements of the study design early in the article. Consider including a table listing sources of data for all phases of the study. For each data source contributing to the analysis, describe the following: |  |  |
|  | a) | Setting: Describe the study design and the underlying population, if possible. Describe the setting, locations, and relevant dates, including periods of recruitment, exposure, follow-up, and data collection, when available. | Supp methods | “UK Biobank UKB is a prospective cohort study which recruited 500,000 adults from the United Kingdom, aged between 40 and 69 years at a baseline visit which took place between 2006-2010 (1). The participants have undergone comprehensive genetic and physical phenotyping (http://biobank.ctsu.ox.ac.uk/crystal/). The study is overseen by the Ethics Advisory Committee and received approval from the National Information Governance Board for Health and Social Care and Northwest Multi-Centre Research Ethics Committee (11/NW/0382), all participants provided informed consent for this study. As part of the UKB extended imaging study, which commenced in 2014, a number of imaging modalities, including dual-energy X-ray absorptiometry (DXA) scans (iDXA, GE-Lunar) of the hip are being collected (2). Shanghai Changfeng Between 2009 to 2012, a total of 6,595 Chinese participants (over 45 years old) from SC Community were recruited by the SC study (3). Left hip DXAs (iDXA, GE-Lunar) and genetic information were collected in 5,310 participants which met imaging and genotyping quality control. Ethics approval was granted by the ethics committee of Zhongshan Hospital affiliated to Fudan University and written informed consent was provided by all participants.” |
|  | b) | Participants: Give the eligibility criteria, and the sources and methods of selection of participants. Report the sample size, and whether any power or sample size calculations were carried out prior to the main analysis | 5 | As above and “For each HSM (1-10) (Figure 1) a GWAS meta-analysis was conducted in 43,485 participants (20,580 (47%)/22,905 (53%) male/female) across two studies (UKB n=38,175 & SC n=5,310) (see Supplementary Figures 3-13 for Manhattan plots)” |
|  | c) | Describe measurement, quality control and selection of genetic variants | 16 | “Two-sample MR, which uses genetic proxies as instrumental variables, was conducted to estimate the causal effect of each HSM on hip osteoarthritis and hip fracture using the TwoSampleMR R package (27). Conditionally independent variants from this study (p<5x10^-8^) provided genetic instruments for HSMs.” |
|  | d) | For each exposure, outcome, and other relevant variables, describe methods of assessment and diagnostic criteria for diseases | 16 | “For outcomes, hip osteoarthritis data comprised a UKB GWAS of hospital diagnosed hip osteoarthritis, excluding participants included in the present HSM GWAS (19). Hip fracture data comprised a previous GWAS meta-analysis (28) which includes UKB (the 5% sample overlap with the hip shape GWAS is unlikely to bias the MR results (29)).” |
|  | e) | Provide details of ethics committee approval and participant informed consent, if relevant | Supp methods | “The study is overseen by the Ethics Advisory Committee and received approval from the National Information Governance Board for Health and Social Care and Northwest Multi-Centre Research Ethics Committee (11/NW/0382), all participants provided informed consent for this study.”  “Ethics approval was granted by the ethics committee of Zhongshan Hospital affiliated to Fudan University and written informed consent was provided by all participants.” |
| 5 | **Assumptions** | Explicitly state the three core IV assumptions for the main analysis (relevance, independence and exclusion restriction) as well assumptions for any additional or sensitivity analysis | Supp methods | “Sensitivity analyses were used to test the conclusion of our Mendelian randomisation analyses. These analyses relax the assumption of no horizontal pleiotropy, assuming uncorrelated pleiotropy (MR-Egger), or relax the assumption about the number of invalid instruments (weighted median, simple mode and weighted mode).“ |
| 6 | **Statistical methods: main analysis** | Describe statistical methods and statistics used |  |  |
|  | a) | Describe how quantitative variables were handled in the analyses (i.e., scale, units, model) | 15 | “The first 10 HSMs (standardized to mean=0, standard deviation (SD)=1) were used as outcomes in GWAS adjusted for age, sex, genotyping chip and the first 20 ancestry principal components in UKB, and age, sex and the first 10 ancestry principal components in SC” |
|  | b) | Describe how genetic variants were handled in the analyses and, if applicable, how their weights were selected | 16 | “Conditionally independent variants from this study (p<5x10^-8^) provided genetic instruments for HSMs”  “Effect estimates represent a one SD increase for continuous exposures (i.e. HSM) and a doubling of odds for binary exposures (i.e. hip osteoarthritis and fracture).“ |
|  | c) | Describe the MR estimator (e.g. two-stage least squares, Wald ratio) and related statistics. Detail the included covariates and, in case of two-sample MR, whether the same covariate set was used for adjustment in the two samples | 16 | “The inverse-variance weighted (IVW) method was used as the primary analysis, where the causal estimate is obtained by combining the SNP-specific Wald ratios using a random-effects inverse-variance weighted meta-analysis. Strong evidence of a causal association was considered based on a Bonferroni adjusted P<0.005 (as 10 HSMs tested). Sensitivity analyses to test the robustness of our estimates, included weighted median and mode, simple mode and Egger regression (12) (Supplementary Methods). Reverse MR was applied to understand the causal effect of a genetic predisposition of hip osteoarthritis and hip fracture on HSMs” |
|  | d) | Explain how missing data were addressed |  | Not applicable |
|  | e) | If applicable, indicate how multiple testing was addressed | 16 | “Strong evidence of a causal association was considered based on a Bonferroni adjusted P<0.005 (as 10 HSMs tested).” |
| 7 | **Assessment of assumptions** | Describe any methods or prior knowledge used to assess the assumptions or justify their validity | 3 | “Null findings in MR studies, such as our previous study of cam morphology, may reflect the lack of suitably powered genetic instruments. One way to assess the precision and power of genetic instruments for an exposure such as hip shape is to examine their relationships with another disease for which a causal effect is thought to exist. If a causal effect is found between the genetic instruments and one condition but not another, this makes it less likely the null findings result from inaccurate or under-powered genetic instruments. Hip fracture provides a good positive control for hip osteoarthritis given that measures of hip geometry, such as femoral neck width, are well established causal risk factors for hip fracture in both observational (7) and MR studies (22).” |
| 8 | **Sensitivity analyses and additional analyses** | Describe any sensitivity analyses or additional analyses performed (e.g. comparison of effect estimates from different approaches, independent replication, bias analytic techniques, validation of instruments, simulations) | 16 & Supp methods | “Sensitivity analyses to test the robustness of our estimates, included weighted median and mode, simple mode and Egger regression (12)”  “Sensitivity analyses were used to test the conclusion of our Mendelian randomisation analyses. These analyses relax the assumption of no horizontal pleiotropy, assuming uncorrelated pleiotropy (MR-Egger), or relax the assumption about the number of invalid instruments (weighted median, simple mode and weighted mode).“ |
| 9 | **Software and pre-registration** |  |  |  |
|  | a) | Name statistical software and package(s), including version and settings used | 16 | “TwoSampleMR R package” |
|  | b) | State whether the study protocol and details were pre-registered (as well as when and where) |  | Not applicable |
|  | **RESULTS** |  |  |  |
| 10 | **Descriptive data** |  |  |  |
|  | a) | Report the numbers of individuals at each stage of included studies and reasons for exclusion. Consider use of a flow diagram | 5 | “For each HSM (1-10) (Figure 1) a GWAS meta-analysis was conducted in 43,485 participants (20,580 (47%)/22,905 (53%) male/female) across two studies (UKB n=38,175 & SC n=5,310)” |
|  | b) | Report summary statistics for phenotypic exposure(s), outcome(s), and other relevant variables (e.g. means, SDs, proportions) | 24 | Table 1 |
|  | c) | If the data sources include meta-analyses of previous studies, provide the assessments of heterogeneity across these studies | Supp results | Supplementary Tables 1-10 include heterogeneity statistics for the GWAS |
|  | d) | For two-sample MR:  i.  Provide justification of the similarity of the genetic variant-exposure associations between the exposure and outcome samples  ii.  Provide information on the number of individuals who overlap between the exposure and outcome studies | 16 | “For outcomes, hip osteoarthritis data comprised a UKB GWAS of hospital diagnosed hip osteoarthritis, excluding participants included in the present HSM GWAS (19). Hip fracture data comprised a previous GWAS meta-analysis (28) which includes UKB (the 5% sample overlap with the hip shape GWAS is unlikely to bias the MR results (29)).” |
| 11 | **Main results** |  |  |  |
|  | a) | Report the associations between genetic variant and exposure, and between genetic variant and outcome, preferably on an interpretable scale | Supp results | Full GWAS results for all associated variants are included in Supplementary Tables 1-10. |
|  | b) | Report MR estimates of the relationship between exposure and outcome, and the measures of uncertainty from the MR analysis, on an interpretable scale, such as odds ratio or relative risk per SD difference | 8 | “MR analyses suggested there was no causal effect of any HSM on hip osteoarthritis (Figure 3, Supplementary Table 18).…. Genetic predisposition to hip osteoarthritis was suggested to causally effect HSM3 (cam-type femoral head with bulging of the lateral aspect) (IVW β 1.37 [95% CI 0.54-2.20], P 1.21×10^-3^) (Figure 3, Supplementary Table 19). Sensitivity analyses showed the same direction of effect but with weaker statistical evidence (Supplementary Table 19).  Counter to the results seen for hip osteoarthritis, MR analyses showed strong evidence for HSM2 (more obtuse neck-shaft angle and less acetabular coverage, as represented by the +2 SD line (Figure 1)) and HSM4 (less acetabular coverage and wider femoral neck, as represented by the -2 standard deviation (SD) line (Figure 1)) having a causal effect on hip fracture (IVW OR 1.27 [95% CI 1.12-1.44], P 1.79×10^-4^ and OR 0.74 [0.65-0.84], P 7.60×10^-6^ respectively) (Figure 3, Supplementary Table 20). These effects were broadly supported by the sensitivity analyses (Supplementary Table 20). There was weaker evidence that HSM3 (a cam-type femoral head and shorter femoral neck) may also have a causal effect on hip fracture (IVW OR 1.16 [95% CI 1.02-1.32], P 0.02] but the sensitivity analyses showed little evidence to support this. There was little evidence that a genetic predisposition to hip fracture affects any of the HSM modes (Figure 3 Supplementary Table 20)” |
|  | c) | If relevant, consider translating estimates of relative risk into absolute risk for a meaningful time period |  | Not applicable |
|  | d) | Consider plots to visualize results (e.g. forest plot, scatterplot of associations between genetic variants and outcome versus between genetic variants and exposure) |  | Figure 3, Supplementary figure 14 |
| 12 | **Assessment of assumptions** |  |  |  |
|  | a) | Report the assessment of the validity of the assumptions | 8 | “The mean F-statistics for the genetic instruments of the HSMs ranged from 37 to 56 (Supplementary Tables 1-10) indicating acceptable instrument strength (12). Reverse MR used 27 genetic instruments for hip osteoarthritis, which had a mean F-statistic of 45 (range 30-100), indicating acceptable instrument strength.” |
|  | b) | Report any additional statistics (e.g., assessments of heterogeneity across genetic variants, such as *I^2^*, Q statistic or E-value) | Supp results | Supplementary Tables 1-10 include *I^2^* |
| 13 | **Sensitivity analyses and additional analyses** |  |  |  |
|  | a) | Report any sensitivity analyses to assess the robustness of the main results to violations of the assumptions | Supp results | Supplementary Tables 19-20 |
|  | b) | Report results from other sensitivity analyses or additional analyses | Supp results | Supplementary Tables 19-20 |
|  | c) | Report any assessment of direction of causal relationship (e.g., bidirectional MR) | Supp results | Supplementary Tables 19-20 |
|  | d) | When relevant, report and compare with estimates from non-MR analyses |  | Not applicable |
|  | e) | Consider additional plots to visualize results (e.g., leave-one-out analyses) |  | Figure 3 |
|  | **DISCUSSION** |  |  |  |
| 14 | **Key results** | Summarize key results with reference to study objectives | 11 | “MR analyses failed to show evidence of a causal effect of hip shape on hip osteoarthritis but did show a causal effect of hip shape, in the form of a more obtuse neck shaft angle and reduced acetabular coverage, on hip fracture. In addition, there was evidence that a genetic predisposition to osteoarthritis caused a cam-type femoral head to develop.” |
| 15 | **Limitations** | Discuss limitations of the study, taking into account the validity of the IV assumptions, other sources of potential bias, and imprecision. Discuss both direction and magnitude of any potential bias and any efforts to address them | 13-14 | “A limitation of this study is that UKB provides the majority of the participants (88%) and therefore the results are biased towards this population. Further work is justified to replicate these findings within a broader array of studies. A further limitation is the average age of the participants was ~60 years old, potentially leading to the coexistence of osteoarthritis in some individuals. However, the majority of participants did not exhibit any signs of radiological osteoarthritis (~90%), and all outline points, which were placed around the contour of the bones, were manually checked to make sure they did not encompass osteophytes (47). Even if osteophytes were inadvertently included in our measures of hip shape, one would expect this to lead to false positive results from MR rather than the null associations found. Another limitation is the use of European reference panels for COJO and genetic correlation analyses that contained East Asian participants, which might result in biased estimates. That said, other ancestrally diverse GWAS meta-analyses have used European reference panel when conducting COJO with promising results (48). Finally, there are known sex differences in hip shape and this study did not examine the X chromosome. Further work is needed to understand X chromosome based genetic pathways involved in the development of hip shape.“ |
| 16 | **Interpretation** |  |  |  |
|  | a) | Meaning: Give a cautious overall interpretation of results in the context of their limitations and in comparison with other studies | 12 | “There was little evidence that any HSM had a causal effect on hip osteoarthritis despite a previous study using the same participants in UKB showing strong observational associations between these HSMs and hip osteoarthritis and total hip replacement (21). These results counter the hypothesis that moderate alterations in hip shape (e.g. cam morphology or acetabular dysplasia) cause hip osteoarthritis in the general population (17, 38). However, they align with our recent study, in the same participants, which found no strong evidence of a causal association between cam morphology (based on alpha-angle derived from the same points used to generate HSMs in the present study) and hip osteoarthritis. Importantly the present study had greater power to explore these associations due to higher number of genetic instruments for each HSM (apart from HSM5) as compared to the alpha-angle study which had only 8 genetic instruments (19).” |
|  | b) | Mechanism: Discuss underlying biological mechanisms that could drive a potential causal relationship between the investigated exposure and the outcome, and whether the gene-environment equivalence assumption is reasonable. Use causal language carefully, clarifying that IV estimates may provide causal effects only under certain assumptions | 12 | “The independent SNPs associated with each HSM were used as genetic proxies for the described hip shape variation in Mendelian randomisation, to estimate the causal effect of each HSM on hip osteoarthritis and fracture. There was little evidence that any HSM had a causal effect on hip osteoarthritis despite a previous study using the same participants in UKB showing strong observational associations between these HSMs and hip osteoarthritis and total hip replacement (21). These results counter the hypothesis that moderate alterations in hip shape (e.g. cam morphology or acetabular dysplasia) cause hip osteoarthritis in the general population (17, 38). However, they align with our recent study, in the same participants, which found no strong evidence of a causal association between cam morphology (based on alpha-angle derived from the same points used to generate HSMs in the present study) and hip osteoarthritis. Importantly the present study had greater power to explore these associations due to higher number of genetic instruments for each HSM (apart from HSM5) as compared to the alpha-angle study which had only 8 genetic instruments (19). Interestingly, in the previous cam morphology MR study there was strong evidence that a genetic predisposition to hip osteoarthritis caused cam morphology and in the present study the same was seen with HSM3 which resembles a cam-type hip with a bulging of the lateral aspect of the femoral head. This adds evidence to the hypothesis that in part osteoarthritis results from a recapitulation of dysregulated growth (39). The shared genetic loci and correlations between hip shape and hip osteoarthritis considered alongside these MR findings suggest that other hip shapes such as reduced or increased acetabular coverage represented by HSM1&2 (i.e. acetabular dysplasia and pincer morphology) might have shared underlying genetics with hip osteoarthritis but do not appear to cause hip osteoarthritis.” |
|  | c) | Clinical relevance: Discuss whether the results have clinical or public policy relevance, and to what extent they inform effect sizes of possible interventions | 12 | “Our results suggest that, at least within the context of the older adult population studied here, changes in hip shape are unlikely to play a causal role in the development of hip osteoarthritis. These findings have implications for treatments targeting hip shape with a view to delaying onset or slowing the progression of hip osteoarthritis in older adults including surgery that are currently being investigated in randomised trials (18, 40)” |
| 17 | **Generalizability** | Discuss the generalizability of the study results (a) to other populations, (b) across other exposure periods/timings, and (c) across other levels of exposure | 13 | “The majority of previous hip shape studies have focused on European populations, but in this study we included an East Asian ancestry group, which broadens the generalisability of our findings (7, 42). However, conducting trans-ancestry GWAS introduces complexity, due to the inherent heterogeneity between populations (43). In this study we used a conservative random-effects model to take account of this heterogeneity and limit the possibility of spurious false positive results driven by the larger size of UKB.” |
|  | **OTHER INFORMATION** |  |  |  |
| 18 | **Funding** | Describe sources of funding and the role of funders in the present study and, if applicable, sources of funding for the databases and original study or studies on which the present study is based | 18 | “BGF is supported by a NIHR Academic Clinical Lectureship and was previously supported by a Medical Research Council (MRC) Clinical Research Training Fellowship (MR/S021280/1). RE, MF, FS were supported, and this work is funded by a Wellcome Trust collaborative award (209233/Z/17/Z). MF conducted this work whilst working at the University of Bristol but is now employed by Boehringer Ingleheim UK and Ireland. CL is funded by a Sir Henry Dale Fellowship jointly funded by the Wellcome Trust and the Royal Society (223267/Z/21/Z). This research was funded in whole, or in part, by the Wellcome Trust [Grant numbers 080280/Z/06/Z, 20378/Z/16/Z, 223267/Z/21/Z]. For the purpose of open access, the authors have applied a creative commons attribution license (CC BY) public copyright licence to any Author Accepted Manuscript version arising from this submission. NCH acknowledges support from the Medical Research Council (MRC) [MC_PC_21003; MC_PC_21001] and National Institute for Health and Care Research (NIHR) Southampton Biomedical Research Centre, University of Southampton and University Hospital Southampton NHS Foundation Trust, Southampton, UK. JPK is funded by a National Health and Medical Research Council (Australia) Investigator grant (GNT1177938). SW is supported by the “Strategic Priority Research Program” of the Chinese Academy of Sciences (Grant No. XDB38020400) and Shanghai Municipal Science and Technology Major Project, Grant No.2017SHZDZX01. XG is supported by Shanghai Municipal Science and Technology Major Project (Grant No. 2017SHZDZX01).“ |
| 19 | **Data and data sharing** | Provide the data used to perform all analyses or report where and how the data can be accessed, and reference these sources in the article. Provide the statistical code needed to reproduce the results in the article, or report whether the code is publicly accessible and if so, where | 19 | “The HSM GWAS meta-analysis summary statistics will be uploaded to the GWAS catalog (<https://www.ebi.ac.uk/gwas/>). The individual level data from this study concerning UKB participants is available via their data showcase. Users must be registered with UK Biobank to access their resources (https://bbams.ndph.ox.ac.uk/ams/).” |
| 20 | **Conflicts of Interest** | All authors should declare all potential conflicts of interest | 18 | “CL has a patent for an image processing apparatus and method for fitting a deformable shape model to an image using random forest regression voting. This is licensed with royalties to Optasia Medical. NH reports consultancy fees and honoraria from Amgen, UCB, Kyowa Kirin, Theramex” |

This checklist is copyrighted by the Equator Network under the Creative Commons Attribution 3.0 Unported (CC BY 3.0) license.

1. Skrivankova VW, Richmond RC, Woolf BAR, Yarmolinsky J, Davies NM, Swanson SA, et al. Strengthening the Reporting of Observational Studies in Epidemiology using Mendelian Randomization (STROBE-MR) Statement. JAMA. 2021;under review.

2. Skrivankova VW, Richmond RC, Woolf BAR, Davies NM, Swanson SA, VanderWeele TJ, et al. Strengthening the Reporting of Observational Studies in Epidemiology using Mendelian Randomisation (STROBE-MR): Explanation and Elaboration. BMJ. 2021;375:n2233.
